# Prelamin A mediates myocardial inflammation in dilated and HIV-associated cardiomyopathies

**DOI:** 10.1172/jci.insight.126315

**Published:** 2019-11-14

**Authors:** Daniel Brayson, Andrea Frustaci, Romina Verardo, Cristina Chimenti, Matteo Antonio Russo, Robert Hayward, Sadia Ahmad, Gema Vizcay-Barrena, Andrea Protti, Peter S. Zammit, Cristobal G. dos Remedios, Elisabeth Ehler, Ajay M. Shah, Catherine M. Shanahan

**Affiliations:** 1 School of Cardiovascular Medicine and Sciences, King’s College London BHF Centre for Research Excellence, London, United Kingdom.; 2 Department of Cardiovascular, Nefrologic, Anestesiologic and Geriatric Sciences, La Sapienza University of Rome, Italy.; 3 National Institute for Infectious Diseases IRCCS “L. Spallanzani”, Rome, Italy.; 4 MEBIC Open University San Raffaele and IRCCS San Raffaele Pisana, Laboratory of Molecular and Cellular Pathology, Milan, Italy.; 5 Centre for Ultrastructural Imaging, Guy’s Campus, and; 6 Randall Centre for Cell and Molecular Biophysics, King’s College London, London, United Kingdom.; 7 Department of Anatomy, Bosch Institute, University of Sydney, Sydney, Australia.

**Keywords:** AIDS/HIV, Cardiology, Cardiovascular disease

## Abstract

Cardiomyopathies are complex heart muscle diseases that can be inherited or acquired. Dilated cardiomyopathy can result from mutations in *LMNA,* encoding the nuclear intermediate filament proteins lamin A/C. Some *LMNA* mutations lead to accumulation of the lamin A precursor, prelamin A, which is disease causing in a number of tissues, yet its impact upon the heart is unknown. Here, we discovered myocardial prelamin A accumulation occurred in a case of dilated cardiomyopathy, and we show that a potentially novel mouse model of cardiac-specific prelamin A accumulation exhibited a phenotype consistent with inflammatory cardiomyopathy, which we observed to be similar to HIV-associated cardiomyopathy, an acquired disease state. Numerous HIV protease therapies are known to inhibit ZMPSTE24, the enzyme responsible for prelamin A processing, and we confirmed that accumulation of prelamin A occurred in HIV^+^ patient cardiac biopsies. These findings (a) confirm a unifying pathological role for prelamin A common to genetic and acquired cardiomyopathies; (b) have implications for the management of HIV patients with cardiac disease, suggesting protease inhibitors should be replaced with alternative therapies (i.e., nonnucleoside reverse transcriptase inhibitors); and (c) suggest that targeting inflammation may be a useful treatment strategy for certain forms of inherited cardiomyopathy.

## Abstract 2

Accumulation of prelamin A occurs in dilated and HIV associated cardiomyopathy causing inflammation, which could be ameliorated by switching therapies in HIV patients.

## Graphical abstract

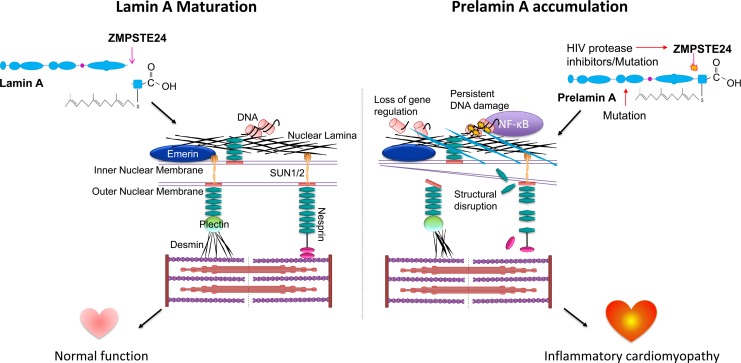

## Introduction

Mutations in the *LMNA* gene are commonly implicated in dilated cardiomyopathy (DCM) phenotypes (1), accounting for approximately 6% of all cases (2). Investigation of in vivo mouse models harboring *LMNA* mutations associated with clinical DCM have identified a number of mechanisms associated with disease (3). However, some questions remain unresolved — in particular, whether the lamin A precursor, prelamin A, is involved in the pathogenesis of cardiomyopathies (4–7).

The *LMNA* gene produces 2 distinct proteins, lamin A and lamin C, which — together with the B-type lamins — form the nuclear lamina that sits adjacent to the inner nuclear membrane (INM) of the nuclear envelope (NE), on the nucleoplasmic side (8). The primary role of the lamina is to provide structural stability to the nuclear environment and to anchor heterochromatin, thereby facilitating appropriate gene expression and efficient DNA damage repair (9, 10). Additionally, the lamina forms part of the linker of nucleoskeleton to cytoskeleton (LINC) complex, which mediates physical communication with the cytoplasmic environment, enabling rapid responses to physical cues, a process termed mechanotransduction (11).

To achieve lamin A maturation, its precursor prelamin A requires step-wise proteolytic processing (12). After translation, addition of farnesyl and carboxymethyl groups to a CAAX motif in the C terminus occurs, followed by an upstream cleavage exclusively mediated by zinc metalloproteinase STE24 homologue, ZMPSTE24, to yield mature lamin A (13–16). Retention of this farnesylated C terminal domain by lamin A precursors is pathologic, and mutations in both the *LMNA* and *ZMPSTE24* genes that cause this retention are implicated in premature aging disorders, such as Hutchinson-Gilford progeria syndrome (HGPS) and DCM. HGPS patients develop cardiomegaly, atrial enlargement, and age-dependent diastolic and systolic dysfunction and left ventricle (LV) hypertrophy (17–19), while the DCM-causing mutation *LMNA*-R89L has been shown to result in aberrant processing and accumulation of prelamin A (5, 7). Moreover, a mutation in *LMNA* postulated to inhibit prelamin A processing, which causes Dunnigan-type familial lipodystrophy, is also associated with cardiac dysfunction; patients homozygous for this mutation have worse LV function, indicating a dose-dependent effect (4). Additionally, a mutation in *ZMPSTE24* known to confer a reduction in enzyme activity was found in a patient with metabolic syndrome and cardiomyopathy (6).

Another cause of prelamin A accumulation is via the pharmacological inhibition of ZMPSTE24 activity by HIV protease inhibitors (HIV PIs) used in the treatment of HIV. HIV PIs result in prelamin A accumulation in cells and potentially contribute to adverse effects (20, 21). HIV patients have double the risk for developing cardiovascular disease than noncarriers (22). Moreover, HIV patients can develop HIV-associated cardiomyopathy (23), though the etiology is complex (24). Previous studies have identified nucleoside reverse transcriptase inhibitors (NRTIs) used in conjunction with HIV PIs as responsible for the development of cardiomyopathy in HIV patients (25, 26). Presently, there is limited knowledge on the impact of HIV PIs on the development of cardiomyopathy, though there is an attempt to characterize heart function on antiretroviral therapy with the introduction of the Characterizing Heart Function on Antiretroviral Therapy (CHART) study (27). These points considered, we sought to investigate the extent and effects of prelamin A accumulation in the setting of cardiomyopathy.

## Results

### Prelamin A can accumulate in DCM patient myocardium.

**Figure 1:**
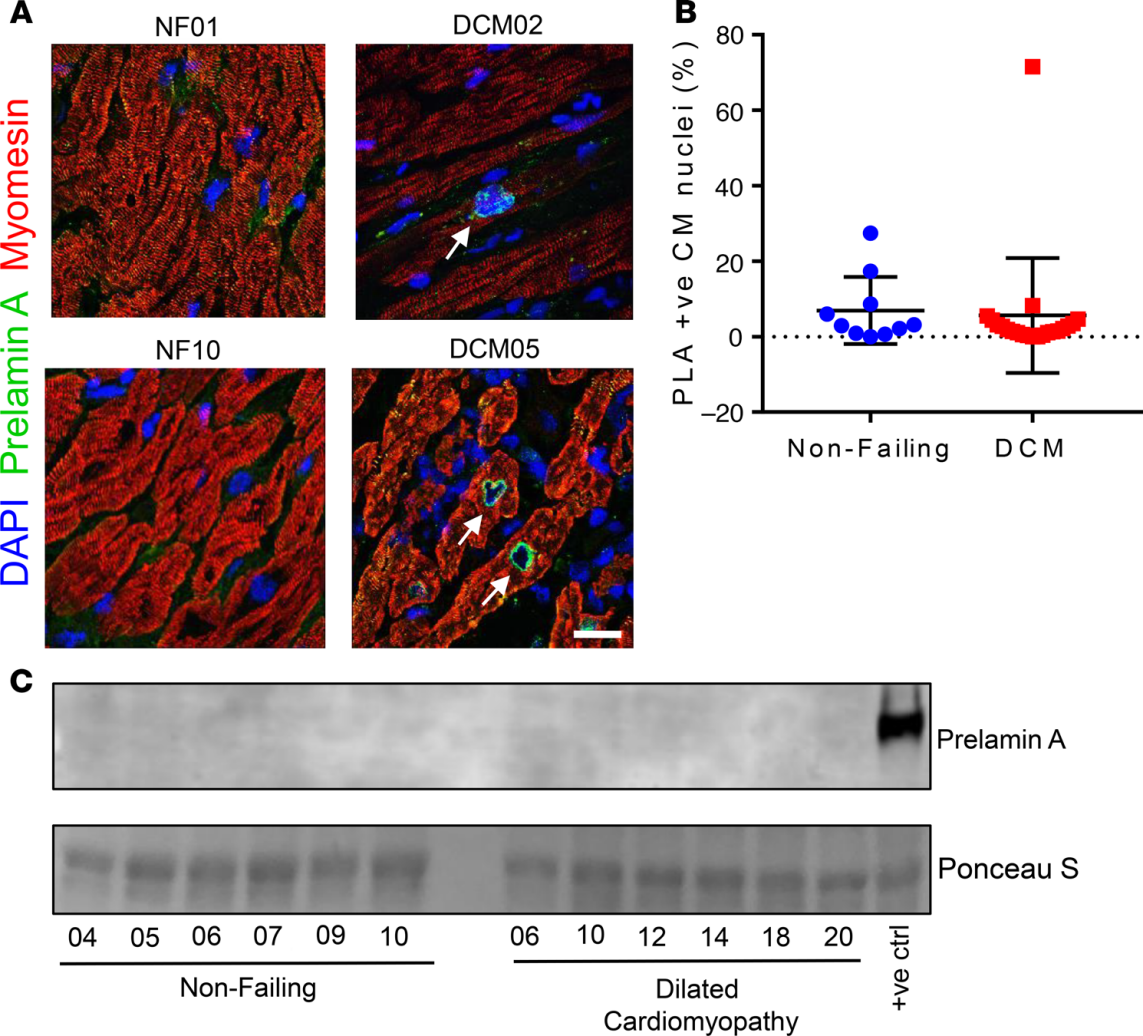
Prelamin A accumulated in a heart of a patient with dilated cardiomyopathy (DCM). (**A**) Confocal micrographs of human heart sections from DCM patients and nonfailing (NF) controls subjected to immunofluorescence staining to detect prelamin A (green), myomesin (red), and DAPI (blue). Arrows point to prelamin A^+^ CM nuclei, many of which exhibit nuclear morphology defects. Scale bar: 10 μm. (**B**) The number of nuclei that stained positively for prelamin A were quantified as a percentage of CM nuclei for nonfailing (blue circles, *n* = 10 nonfailing donor samples) and DCM (red squares, *n* = 21 samples from explanted DCM hearts) myocardial sections (mean ± SD). (**C**) Western blotting did not detect prelamin A in a selection of DCM patient samples. There was not enough sample remaining from DCM05 to run on a Western blot.

Immunofluorescence staining for prelamin A was performed on human DCM patient LV samples (Supplemental Table 1; supplemental material available online with this article; https://doi.org/10.1172/jci.insight.126315DS1) and nonfailing (NF) control samples (Supplemental Table 2) and was quantified (Figure 1, A and B). See complete unedited blots in the supplemental material. Sporadic and focal expression of prelamin A was observable in cardiomyocyte (CM) nuclei in both NF and DCM samples. However, in 1 DCM sample (DCM05), consistent CM nuclear rim staining was found in 71.5% of total CM nuclei. Unfortunately, there was no remaining tissue to perform Western blotting on the sample expressing prelamin A; however, Western blotting of a selection of the same samples supported the findings that, generally, prelamin A was not abundant across NF or DCM samples (Figure 1C).

### Prelamin A accumulation in CMs of mice causes cardiomyopathy and premature death by heart failure.

**Figure 2:**
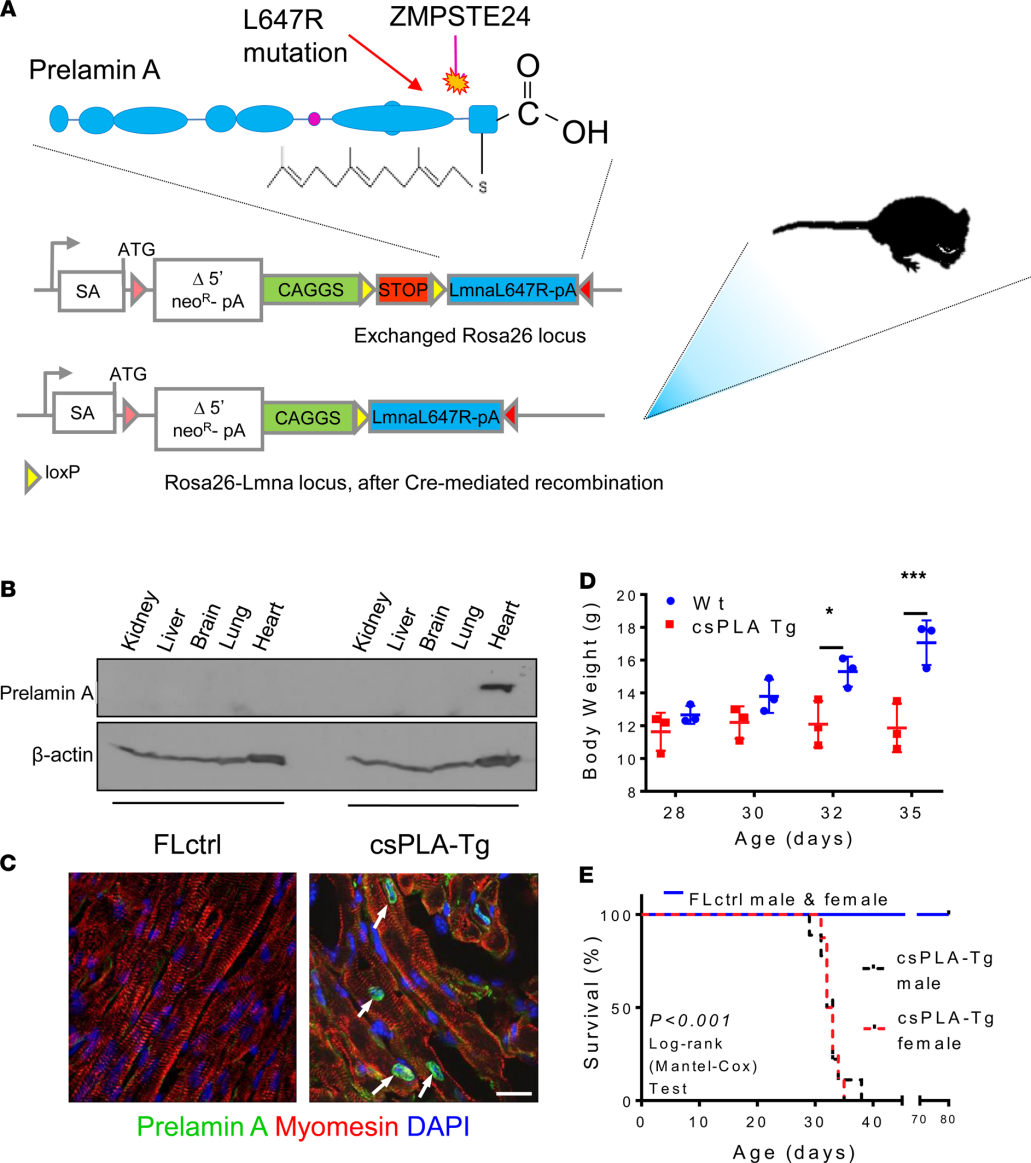
Targeted transgenesis of prelamin A led to nuclear accumulation in CMs and resulted in premature death in mice. (**A**) Schematic representation showing the site of prelamin A (*LMNA*-L647R) cDNA insertion and the modifications required for conditional expression. SA, splice acceptor site; neo^R^, neomycin resistance; pA, polyadenylation signal. (**B**) Western blotting for prelamin A showing expression was restricted to heart tissue. (**C**) Confocal micrographs of myocardium stained for prelamin A showing nuclear rim localization in csPLA-Tg hearts. Scale bar: 10 μm. Arrows indicate prelamin A expressing nuclei. (**C**) Growth curves showing that csPLA-Tg mice stop growing after 30 days. *n* = 3 males/group. Two-way ANOVA with repeated measures with Sidak’s post hoc test for multiple comparisons was performed. *P < 0.05, ***P < 0.001. (**D**) Kaplan-Meier survival analysis showing that csPLA-Tg male and female mice exhibited attenuated survival compared with FLctrl counterparts. *n* = 7 FLctrl males, 8 FLctrl females, 9 csPLA-Tg males, 8 csPLA-Tg females. Log-rank Mantel-Cox test was performed. *P* < 0.0001.

To examine the effects of prelamin A on the heart, CM-specific prelamin A transgenic (csPLA-Tg) mice were generated. csPLA-Tg mice (Figure 2A) were born in a normal Mendelian ratio and were indistinguishable from floxed control (FLctrl) mice at birth. Western blotting confirmed that accumulation of prelamin A in csPLA-Tg mice was specific to the heart (Figure 2B), and immunofluorescence showed that this occurred specifically in the nuclear rim of CMs (Figure 2C).

After weaning (day 21), csPLA-Tg mice ceased to grow and died prematurely. By 32 days, body weight was significantly lower in csPLA-Tg mice (Figure 2D), and median survival was significantly attenuated in male and female mice compared with FLctrl (Figure 2E).

**Figure 3:**
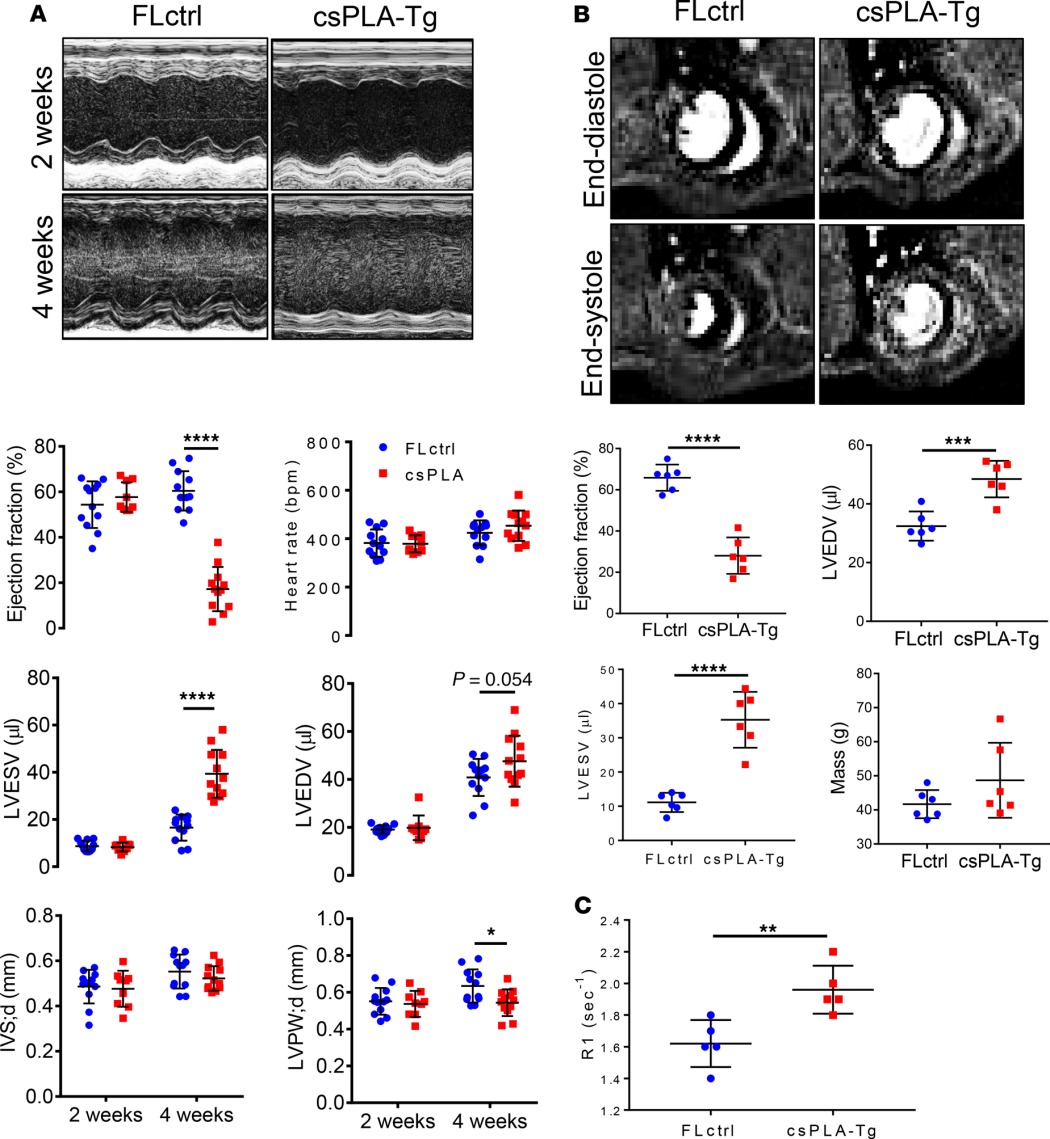
Cardiac function was attenuated in 4-week-old csPLA-Tg mice. (**A**) Representative images of echocardiographs and corresponding graphs of analysis performed on movies acquired in B-mode showing severely compromised cardiac function in 4-week-old mice (*n* = 12/group [6 females, 6 males], except csPLA-Tg 2 weeks, which was *n* = 9 [7 females, 2 males]). Values are mean ± SD. Two-way ANOVA, no repeated measures, was performed with Sidak’s post hoc test for multiple comparisons. *P < 0.05, ****P < 0.0001. (**B**) Representative cardiac MRI images of myocardium in end-systole and end-diastole and corresponding graphs displaying a decrease in ejection fraction alongside increases in left ventricle end-diastolic (LVEDV) and LV end-systolic volume (LVESV). Mass was statistically unchanged, but with increased variation, and concurs with postmortem heart weight measurements. (**C**) Increased relaxation time (R1) of gadolinium contrast in 4-week-old csPLA-Tg myocardium, indicative of fibrosis remodeling. *n* = 6 males/group. Values are mean ± SD. Student’s 2-tailed *t* test was performed. **P < 0.01, ***P < 0.001, ****P < 0.0001. IVS;d, intraventricular septal thickness in diastole; LVPW;d, left ventricle posterior wall thickness in diastole.

At 2 weeks, csPLA-Tg mice showed no change in structural, dimensional, or functional parameters by echocardiography, as compared with FLctrl controls (Figure 3A). In contrast, echocardiographic and MRI analysis of 4-week-old mice showed that there was significant chamber dilatation, as evidenced by increases in LV end-systolic and end-diastolic volumes, as well as significant contractile impairment, as evidenced by a marked reduction in ejection fraction. There was also evidence of LV posterior wall thinning. Heart rates were similar in the 2 groups (Figure 3, A and B). In addition, increased relaxation time of a gadolinium contrast agent in MRI of hearts suggested functionally relevant fibrotic remodeling of the myocardium (Figure 3C).

**Figure 4:**
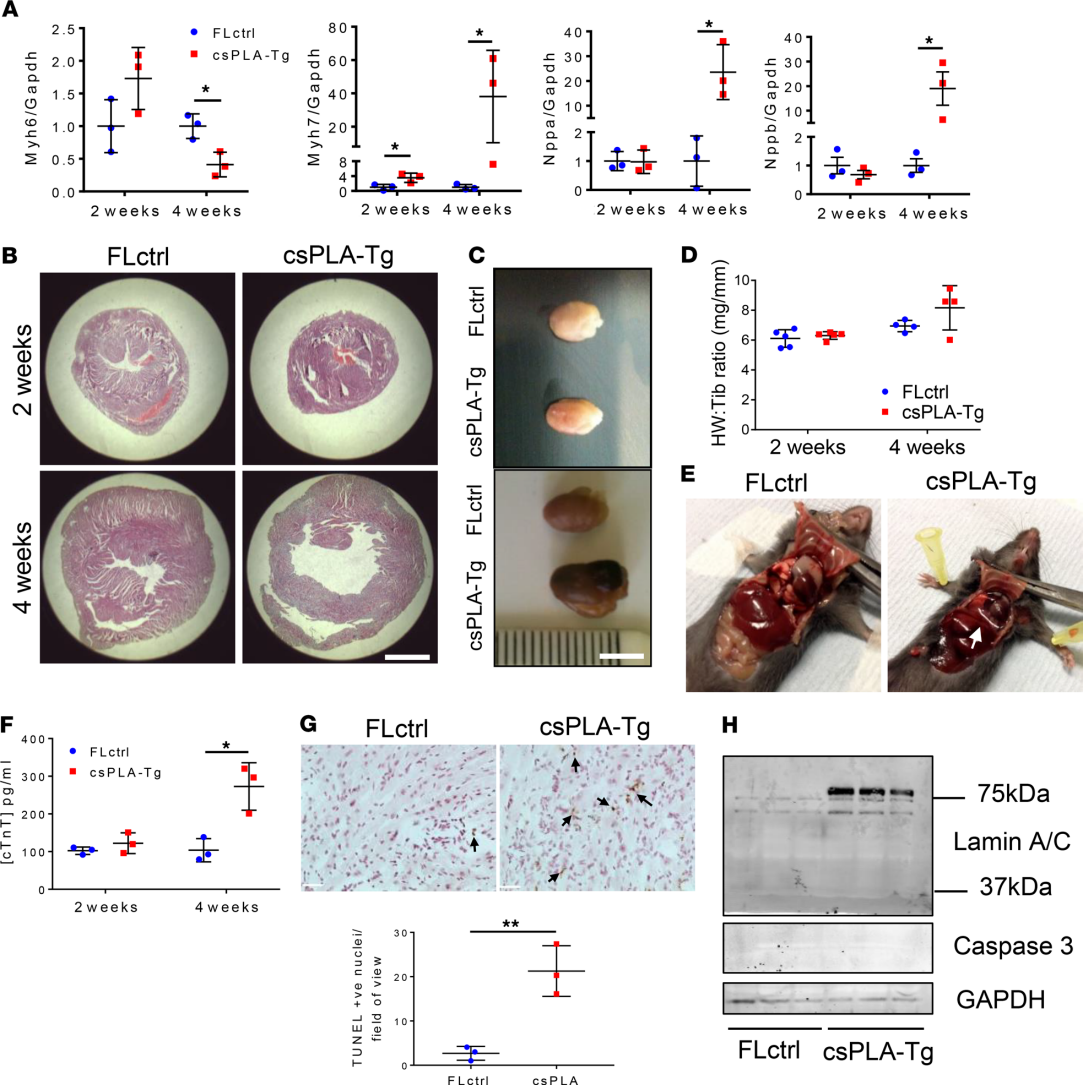
csPLA-Tg mice displayed signs of heart failure and necrotic cell death. (**A**) Fetal gene expression is dysregulated in csPLA-Tg hearts at 4 weeks, indicating heart failure; *n* = 3 females/group. Values are mean ± SD. Two-Way ANVOA, no repeated measures, was performed with Sidak’s post hoc test for multiple comparisons. *P < 0.05. (**B**) Low-magnification micrographs showing cross-sectional view of the heart stained with H&E, indicating 4-week-old csPLA-Tg mice possessed dilated cardiac chambers. Scale bar: 2 mm. (**C**) Photographs showing csPLA-Tg appeared enlarged at 4 weeks. Scale bar: 5 mm. (**D**) No significant difference in heart weight to tibia length ratio was observed. Values are mean ± SD. *n* = 3/group. (**E**) Dissection of chest cavities showing transudative pleural effusions in csPLA-Tg mice. (**F**) Blood plasma subjected to ELISA showed elevated levels of circulating cardiac Troponin T (TnT), indicative of cardiac damage in 4-week-old mice. *n* = 3 females/group. Values are mean ± SD. Two-Way ANOVA, no repeated measures, with Sidak’s post hoc test for multiple comparisons was performed. *P < 0.05. (**G**) TUNEL staining was performed on 4-week-old csPLA-Tg heart sections, and image quantification showed a significant increase in TUNEL^+^ nuclei in csPLA-Tg heart sections at 4 weeks. *n* = 3 females/group. Values are mean ± SD. Student’s 2-tailed *t* test was performed. **P < 0.01. Arrows indicate TUNEL positive nuclei. Scale = 30 µm. (**H**) Western blotting showed that cleavage of caspase 3 and lamins A/C, which occurs at ~37 kDa and occurs during apoptosis, did not occur in 4-week-old csPLA-Tg myocardium.

Quantitative PCR (qPCR) analysis of csPLA-Tg myocardium showed that there was reduced mRNA expression of *Myh6* and increases in *Myh7*, *Nppa*, and *Nppb* mRNA, consistent with heart failure (Figure 4A). This was supported by postmortem analysis, which showed csPLA-Tg hearts were enlarged at 4 weeks (Figure 4, B and C), while mass, based on heart weight relative to tibia length, was similar between csPLA-Tg and FLctrl mice at 2 and 4 weeks (Figure 4D). Transudative pleural effusions were evident upon opening the chest cavity in 4-week-old csPLA-Tg mice symptomatic of heart failure (Figure 4E).

ELISA of blood plasma identified a substantial increase in the plasma concentration of cardiac troponin T in 4-week-old csPLA-Tg mice, indicative of significant CM damage or death (Figure 4F). Increases in TUNEL^+^ nuclei indicated that cell death occurred (Figure 4G), though there was no evidence of caspase 3 cleavage or lamin cleavage, indicators for apoptosis (Figure 4H), suggesting that necrosis rather than apoptosis was the mode of cell death.

### csPLA-Tg myocardium exhibits fibrotic remodeling and an inflammatory senescence-associated secretory phenotype.

**Figure 5:**
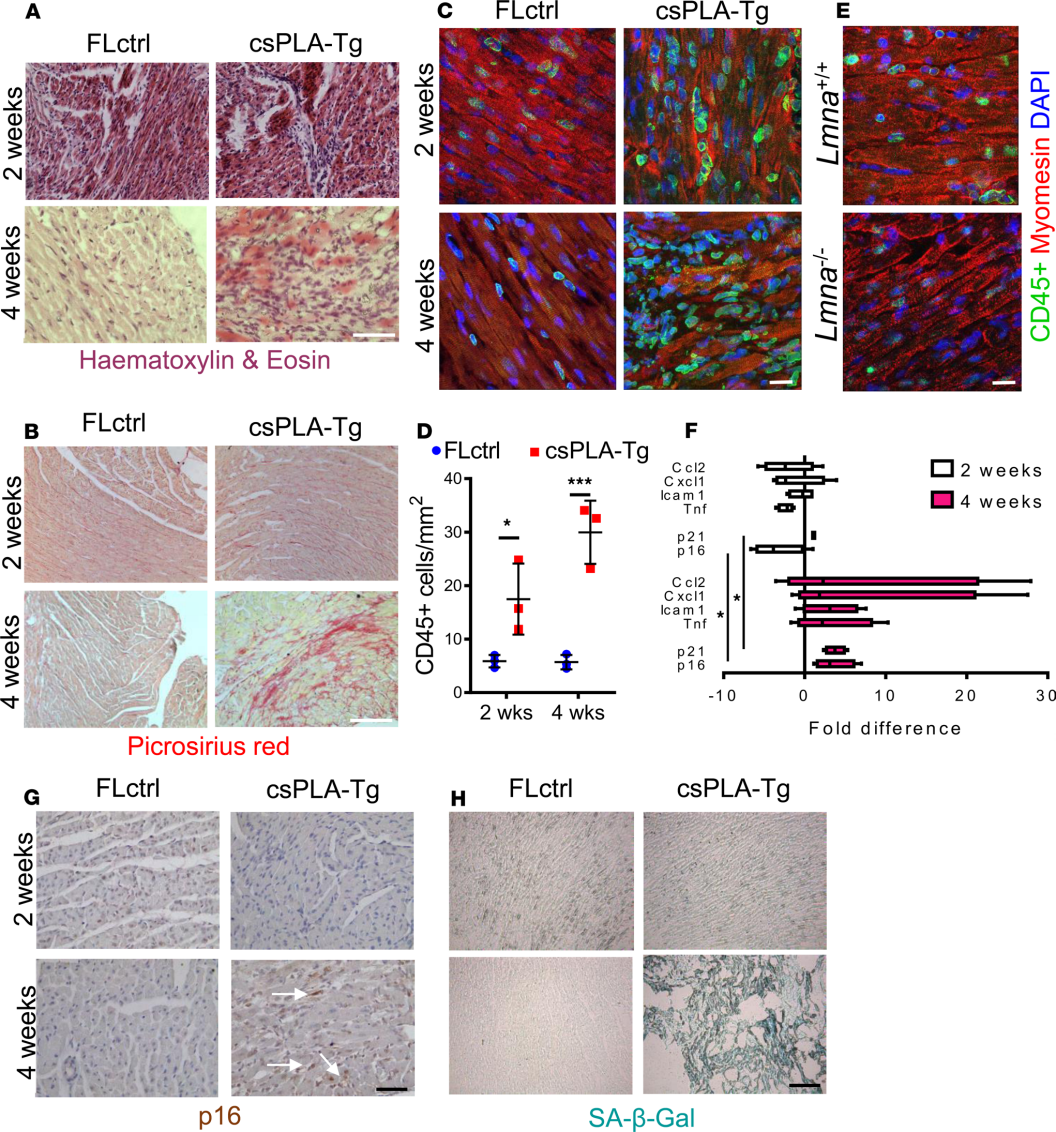
Fibrotic remodeling of csPLA-Tg myocardium occurred in tandem with inflammation and senescence. (**A**) Light micrographs showing myocardial disarray in 4-week-old csPLA-Tg myocardium stained with H&E. Scale bar: 30 μm. (**B**) Light micrographs showing Picrosirius red–stained myocardium to indicate fibrosis in 4-week-old csPLA-Tg myocardium shown by excessive red staining. Scale bar: 30 μm. (**C** and **D**) Quantitative fluorescence immunostaining for CD45 shows presence of CD45^+^ cells in 2- and 4-week-old csPLA-Tg myocardium. Scale bar: 10 μm. Values are mean ± SD. *n* = 3 females/group. Two-way ANOVA with Sidak’s post hoc test for multiple comparisons was performed. *P < 0.05, ***P < 0.001. (**E**) CD45^+^ immunostaining of *Lmna^–/–^*myocardium showed no evidence of infiltration by CD45^+^ leukocyte populations. (**F**) qPCR showing the cytokine profile of csPLA-Tg myocardial mRNA. *n* = 4 females/group. (**G**) Immunohistochemical staining showing expression of p16 in 4-week-old csPLA-Tg myocardium. (**H**) Senescence-associated β-galactosidase was expressed in 4 week csPLA-Tg myocardium. Scale = 30 µm (**G** and **H**).

Inspection of H&E-stained tissue sections showed that csPLA-Tg heart tissue at 2 weeks was mostly normal, with sporadic regions of mononuclear aggregation in the myocardial interstitium. At 4 weeks, however, csPLA-Tg myocardium was in disarray, and substantial mononuclear infiltration was observed (Figure 5A). Similarly, Picrosirius red staining was comparable between csPLA-Tg and FLctrl at 2 weeks, but at 4 weeks, csPLA-Tg myocardium displayed substantial red staining, indicating that fibrotic remodeling had occurred (Figure 5B) and supporting the late gadolinium enhancement (LGE) data observed in the MRI assessment of myocardium. Observation of mononuclear infiltration in the myocardium suggested that inflammatory cells were activated and present in csPLA-Tg hearts, and this was confirmed by immunofluorescence staining of myocardial sections for CD45 (Figure 5, C and D), which showed increased numbers of CD45^+^ cells in the myocardium of both 2- and 4-week-old mice (Figure 5, C and D). Myocardial inflammation has not previously been reported in models of *LMNA* cardiomyopathy, so to test whether this was unique to our model, we performed immunostaining on *Lmna^–/–^* myocardium for CD45 and observed no increase in CD45^+^ cells compared with WT, indicating that this was a feature unique to prelamin A accumulation (Figure 5E). Furthermore, mRNA expression analysis of myocardium for proinflammatory cytokines found that, at 4 weeks, *Tnf*, *Icam1*, *Cxcl1*, and *Ccl2* were elevated in csPLA-Tg myocardium (Figure 5F).

Because disruption to the nuclear lamina is associated with premature senescence (28, 29) and, in turn, senescence is associated with inflammation via the senescence-associated secretory phenotype (SASP) (30), we postulated that csPLA-Tg myocardium may display traits of senescence. This was supported by qPCR, which showed that expression of mRNA for the genes encoding senescence markers p16 *(Cdkn2a*) and p21 (*Cdkn1a*) were upregulated in 4-week-old csPLA-Tg myocardium (Figure 5F). IHC for p16 confirmed an increase at the protein level in 4-week-old csPLA-Tg myocardium (Figure 5G). In addition, intense senescence-associated β-galactosidase (SA–β-gal) staining was observed in 4-week-old csPLA-Tg myocardium when compared with FLctrl (Figure 5H).

**Figure 6:**
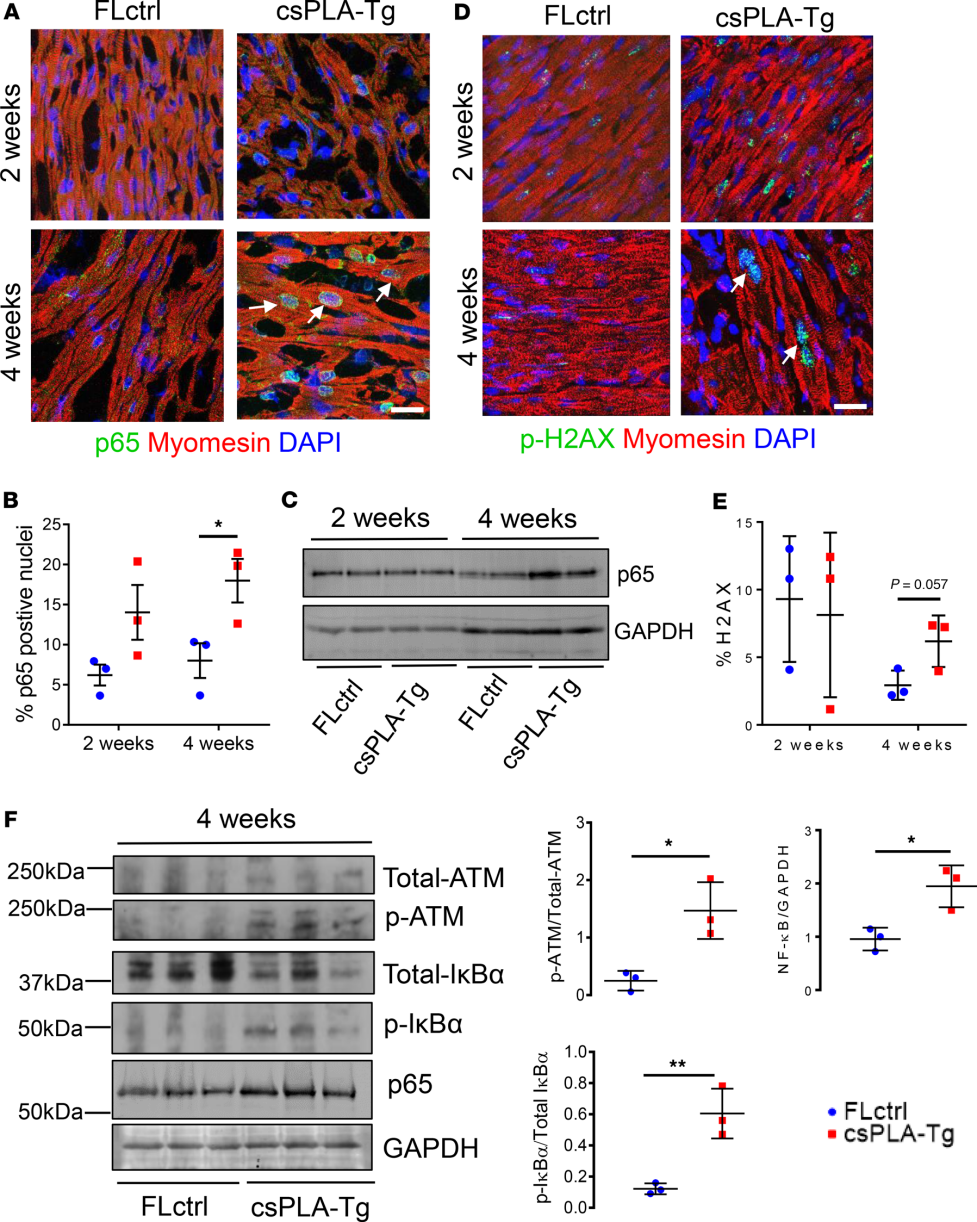
NF-κB signaling was activated in 4-week-old csPLA-Tg CMs and mediated by persistent DNA damage. (**A**) Subcellular localization of p65 subunit of NF-κB in csPLA-Tg myocardium, highlighted by white arrows. (**B**) Quantification of p65 micrographs showing increases in the number of nuclei expressing p65 for 2- and 4-week-old hearts counted as a percentage of total nuclei. *n* = 3 females/group. Values are mean ± SD. *P < 0.05. Two-way ANOVA, no repeated measures, with Sidak’s test for multiple comparisons was performed. (**C**) Western blot showing increase of NF-κB subunit p65 in 4-week-old csPLA-Tg myocardium. (**D**) Confocal micrographs of fluorescence immunostaining showing DNA damage marker γ-H2AX (white arrows). Scale bar: 10 μm. (**E**) Quantification of γ-H2AX micrographs. *n* = 3 females/group. Values are mean ± SD. Two-way ANOVA, no repeated measures, with Sidak’s post hoc test for multiple comparisons was performed. (**F**) Western blots of 4-week-old myocardial lysates showing phosphorylation status of ATM and IκBα and graphs showing corresponding densitometry analyses. Values are mean ± SD. *n* = 3 females/group. Unpaired 2-tailed *t* test was performed. *P < 0.05, **P < 0.001.

*NF-κB signaling was activated in csPLA-Tg myocardium*. NF-κB is a master regulator of inflammation (31). It is also known that prelamin A can activate NF-κB via persistent DNA damage and a noncanonical signaling pathway involving signaling partners such as IκBα (32). Persistent activation of the DNA damage response is also responsible for activation of senescence in laminopathies (9). Thus, we hypothesized that this might be activated in csPLA-Tg mice. The p65 subunit of NF-κB is translocated to the nucleus upon activation; therefore, to investigate this, we performed quantitative immunofluorescence staining and showed that this was occurring at 4 weeks (Figure 6, A and B). To substantiate this finding, Western blot indicated elevated expression of p65 at 4 weeks but not 2 weeks (Figure 6C). We then assessed DNA damage signaling by phosphorylated Histone 2AX (γ-H2AX) — a first responder and activator of DNA damage signaling. We found that γ-H2AX staining as a percentage of total nuclear stain was inconsistent in 2-week-old myocardium, while — in 4-week-old csPLA-Tg myocardium — there was a trend toward an increase (*P* = 0.057) (Figure 6, D and E). To investigate further, we assessed the DNA damage transducer ataxia telangiectasia mutated (ATM), which can activate NF-κB signaling via IκBα. Western blotting of myocardial lysates from 4-week-old mice showed that ATM and IκBα were consistently phosphorylated in csPLA-Tg myocardium (Figure 6F). Taken together, these data infer that activation of inflammatory NF-κB signaling via ATM is a consequence of prelamin A accumulation in the heart.

### Disruption to the LINC complex and cytoskeleton was preceded by loss of histone marks in csPLA-Tg myocardium.

**Figure 7:**
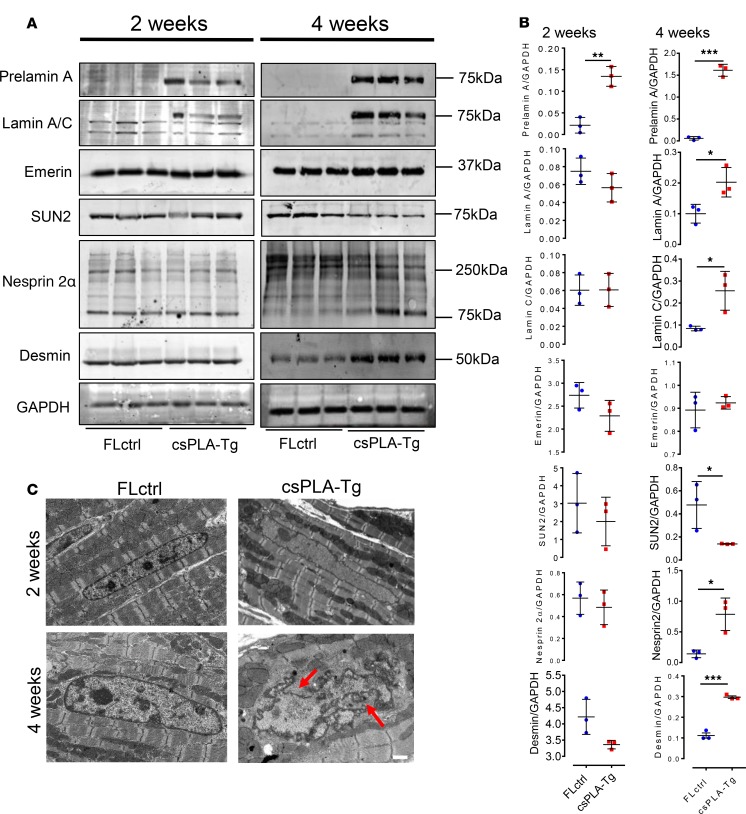
Prelamin A accumulation led to disorganization of molecular structure at 4 weeks and loss of chromatin and histone marks at 2 weeks. (**A** and **B**) Western blot analysis showing the protein expression changes occurring at 4 weeks in structural proteins of the nuclear envelope-lamin A/C, emerin, SUN2, nesprin 2α, and also the cytoskeleton-desmin, with corresponding semiquantitative densitometry analysis. *n* = 3 females/group. Values are mean ± SD. Unpaired 2 tailed *t* test was performed on age-matched groups. Welch’s correction was applied to data for lamin A, lamin C, emerin, SUN2, and nesprin 2α at 4 weeks. *P < 0.05, **P < 0.01, ***P < 0.001. (**C**) Electron micrographs showing nuclear shape and size changes in csPLA-Tg myocardium; red arrows point to regions of nuclear in-folding characteristic of nuclear morphology defects. Scale bar: 1 μm.

**Figure 8:**
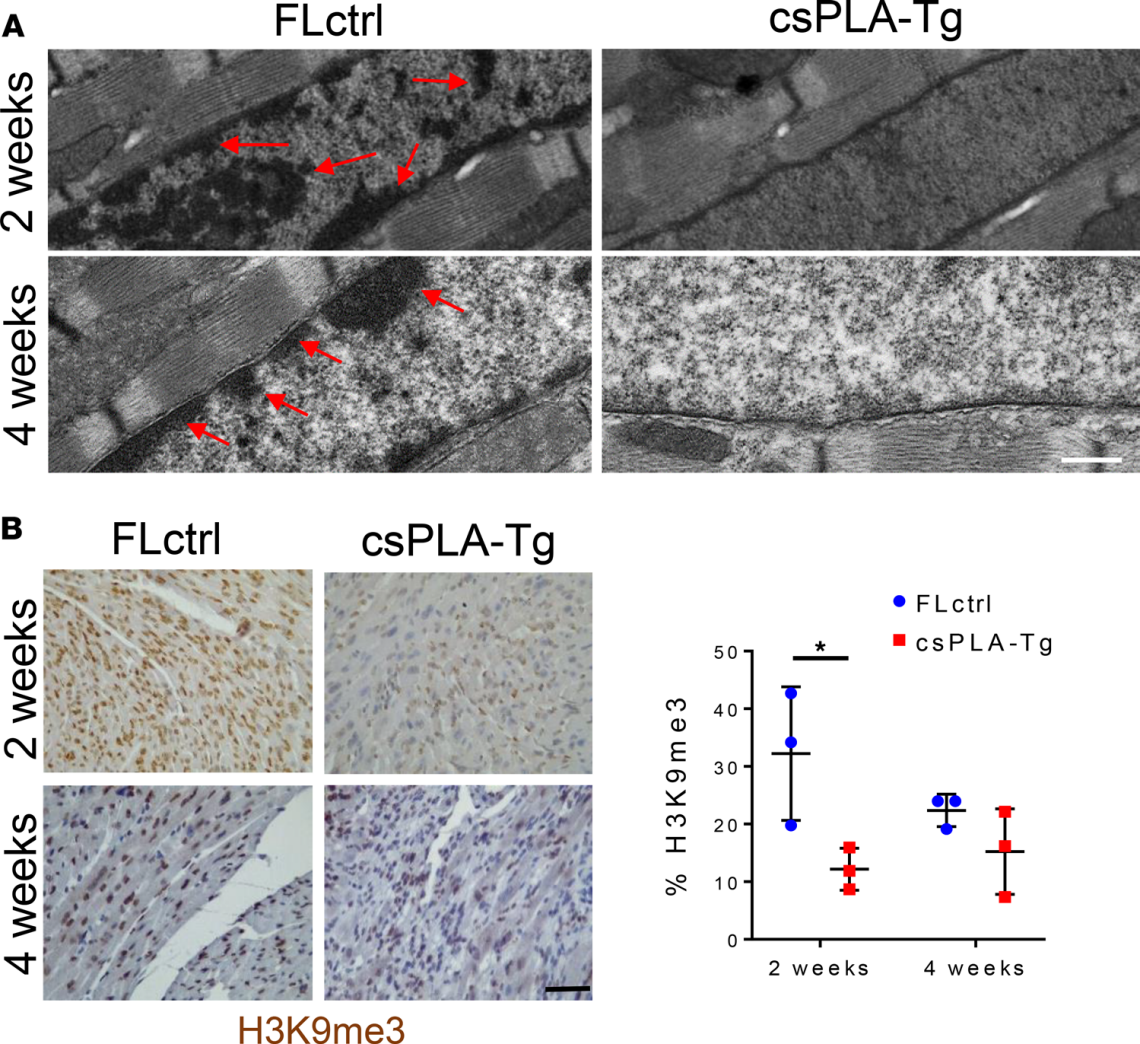
Prelamin A accumulation resulted in loss of heterochromatin and H3K9me3 repressive histone marks in the myocardium of 2-week-old mice. (**A**) Representative electron micrographs show heterochromatin displacement and loss of chromocentres. Scale bar: 500 nm. Arrows indicate regions of condensed chromatin. (**B**) Quantitative IHC showed a profound loss of H3K9me3 staining as a percentage of total Hematoxylin stain in 2-week-old csPLA myocardium. Scale bar: 30 μm. Values are mean ± SD. *n* = 3 females/group. Two-way ANOVA, no repeated measures, with Sidak’s test for multiple comparisons was performed.*P < 0.05.

Inconsistencies between the activation of NF-κB and the onset of inflammation encouraged us to explore other pathways consistently affected in 2-week-old myocardium. The structural hypothesis of lamin dysfunction argues that NE disruption can lead to increased susceptibility to mechanical stress and structural instability of cells (3). Western blotting of LINC complex proteins and the intermediate filament desmin showed profound changes in expression at 4 weeks but not at 2 weeks (Figure 7, A and B). TEM of csPLA-Tg myocardium showed that nuclear morphology defects could be observed at 4 weeks, though not at 2 weeks (Figure 7C). Another theory of lamin dysfunction hypothesis that regulation of gene expression is affected by lamina disruption. TEM images showed a loss of chromocentres and heterochromatin bundles, which did appear to occur at 2 weeks in csPLA-Tg myocardium (Figure 8A). We decided, therefore, to assess the chromatin changes by investigating methylation of lysine 9 of histone 3 (H3K9me3). We performed and quantified immunohistochemical staining for H3K9me3 expression and discovered a profound loss of H3K9me3 in 2-week-old csPLA-Tg myocardium (Figure 8B).

### Prelamin A accumulates in HIV patients undergoing antiretroviral therapy with PIs.

**Figure 9:**
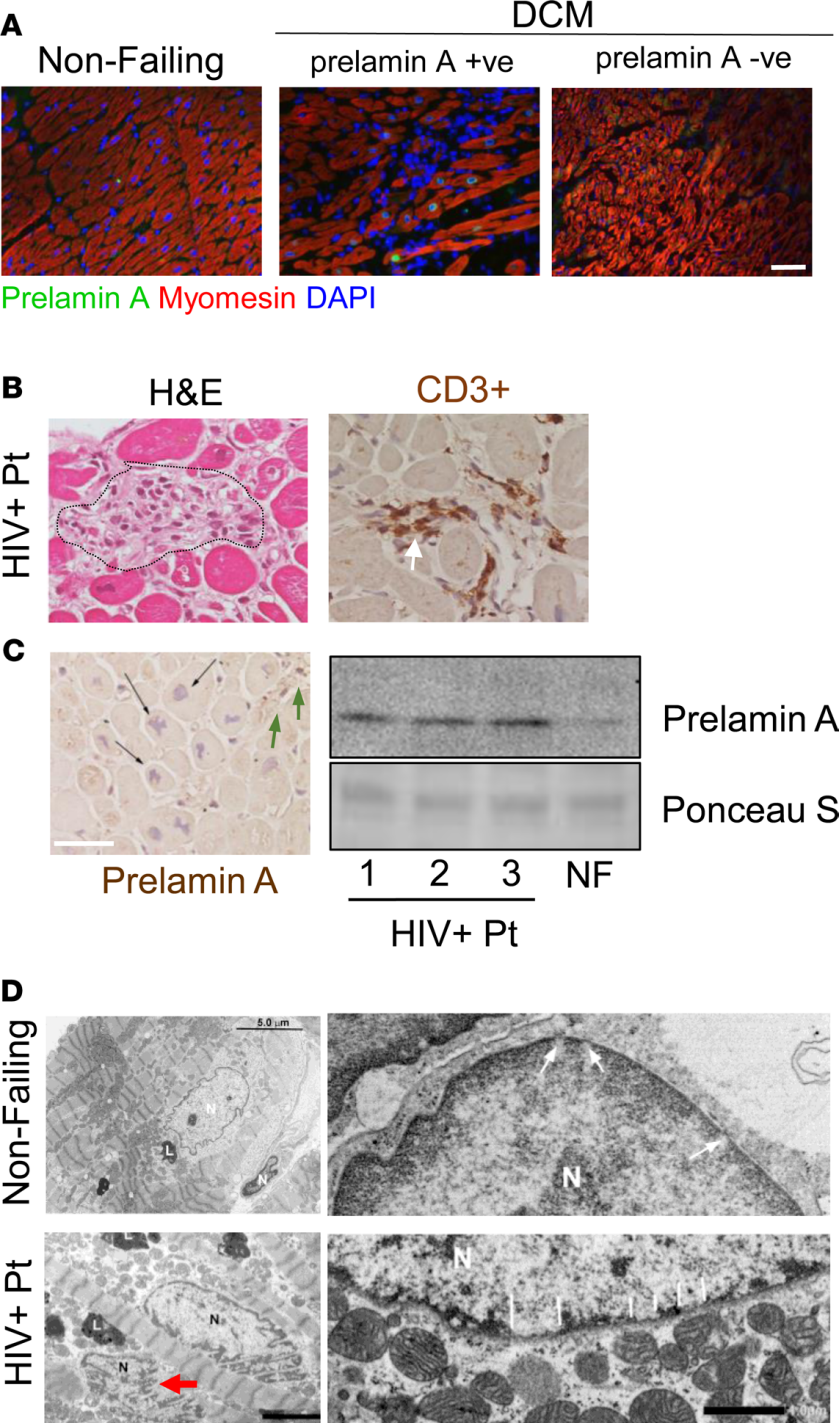
Prelamin A accumulated in hearts of patients with HIV-associated cardiomyopathy under retroviral therapy. **(A**) The DCM patient sample in which prelamin A accumulated showed profound mononuclear infiltration unlike other DCM samples. Scale bar: 40 μm. (**B**) H&E and CD3^+^ IHC showing inflammation in HIV^+^ myocardium consistent with the csPLA-Tg model. Scale bar: 30 μm. (**C**) IHC showing focal prelamin A accumulation in CM nuclei (black arrows) and non-CM populations (green arrows) of HIV^+^ myocardium supported by Western blotting, which detected accumulation of prelamin A in hearts of HIV patients. (**D**) Electron micrographs showing nuclear morphology defects in HIV^+^ myocardium (red arrow; scale bar: 3 μm) and nuclear pore complexes surrounded by evenly spread heterochromatin in nondiseased myocardium (large white arrows) and heterochromatin displacement in HIV^+^ myocardium (small white arrows). Arrows are located at the inner nuclear membrane of lower right panel of **D**. Scale bar: 1 μm. N, nucleus; L, lipid bodies.

Because we observed an inflammatory phenotype in the csPLA-Tg model, we postulated that this may also occur in the human setting. Due to the paucity of material, we were unable to test this with CD45^+^ staining. However, visualization of the prelamin A–stained tissue sample showed regions containing large clusters of nuclei, indicative of leukocyte infiltration, which was not observed to the same extent in non–prelamin A–expressing DCM tissue (Figure 9A). Subsequently, we studied the literature for inflammatory cardiomyopathies and focused on HIV-associated cardiomyopathy (33). Because of the association of HIV PIs with the accumulation of prelamin A in other tissues, we hypothesized that prelamin A would be abundant in HIV^+^ patients on a regime of HIV PIs exhibiting symptoms of cardiomyopathy, as proven by echocardiographic assessment (Table 1). Histological assessment by H&E and IHC for CD3^+^ and prelamin A confirmed the presence of inflammation in conjunction with prelamin A expression (Figure 9B). Indeed, prelamin A IHC showed focal expression of prelamin A in nuclei of CM and non-CM populations within the hearts of HIV^+^ patients, with a number of CM nuclei showing highly aberrant morphologies. Western blotting also revealed that abundance of prelamin A was increased in HIV^+^ patients compared with a NF control (Figure 9C). Aberrant nuclear morphology and changes in the spatial organization of heterochromatin were also observed at the ultrastructural level in HIV^+^ myocardium compared with NF controls (Figure 9D), and these observations were consistent with the nuclear morphology defects and loss of heterochromatin bundles observed by TEM in the csPLA-Tg mice (Figure 7C and Figure 8A).

### Inducible expression of prelamin A in adult hearts causes progressive heart dysfunction, inflammation, and premature death.

**Figure 10:**
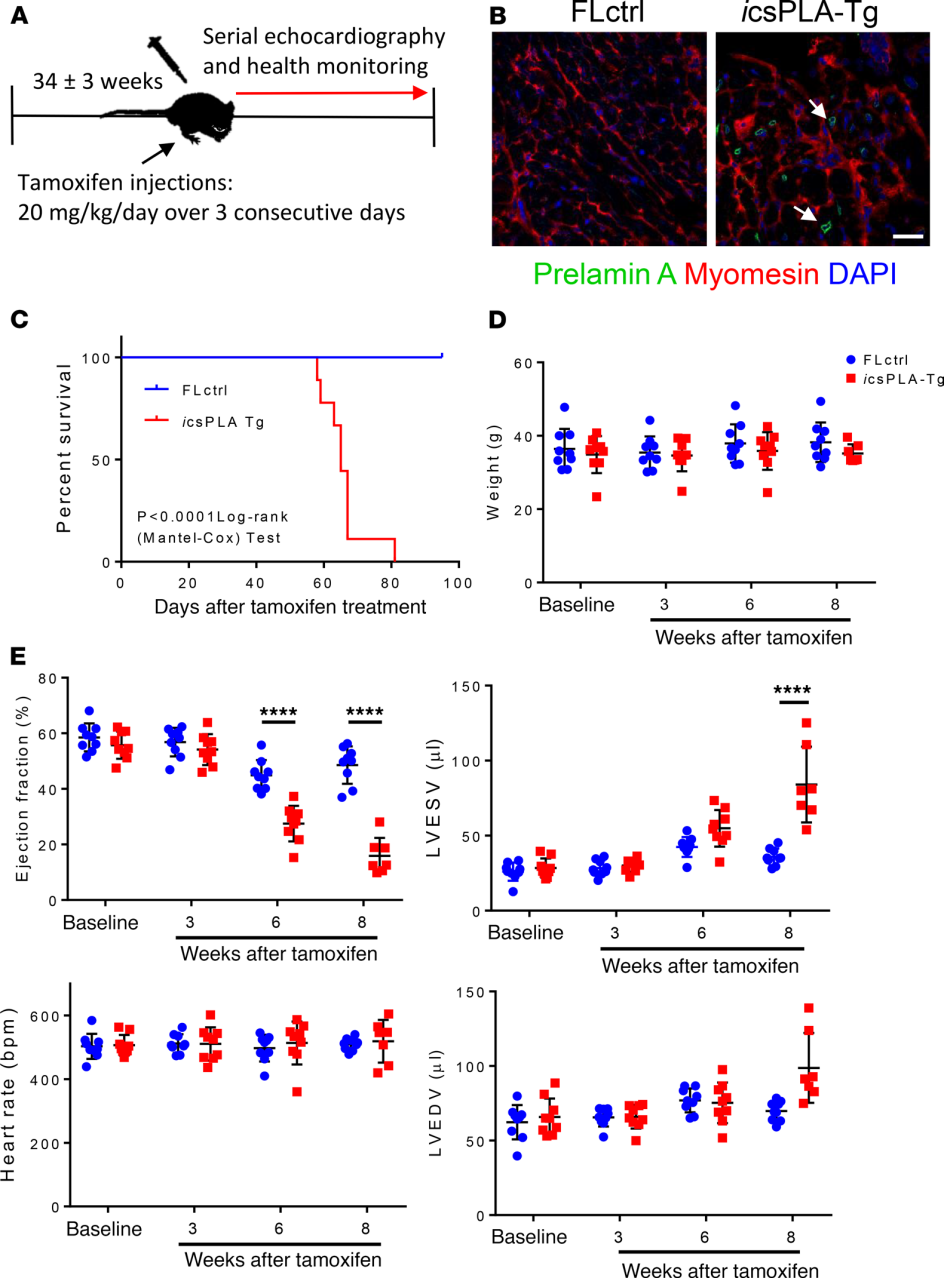
Induction of prelamin A expression in adulthood leads to a progressive and fatal decline in cardiac function in inducible csPLA-Tg (*i*csPLA-Tg) mice. (**A**) Schematic showing the protocol for assessing the effect of prelamin A acquired in adult mouse myocardium via utilization of a tamoxifen inducible MerCreMer promoter. (**B**) Immunofluorescence staining of heart sections showing the expression of prelamin A in cardiomyocytes of *i*csPLA-Tg mice. Scale bar: 20 μm. (**C**) Kaplan-Meier survival analysis showing that csPLA-Tg male and female mice died early compared with FLctrl counterparts. Log-rank Mantel-Cox. *P* < 0.0001. *n* = 9/group. (**D**) Body weights remained constant during the course of the protocol. (**E**) Cardiac function declined progressively over the course of the protocol. Values are mean ± SD. *n* = 9–8 males and 1 female/group. Two-way ANOVA with repeated measures and Sidak’s test for multiple comparisons was performed. ****P < 0.0001.

**Figure 11:**
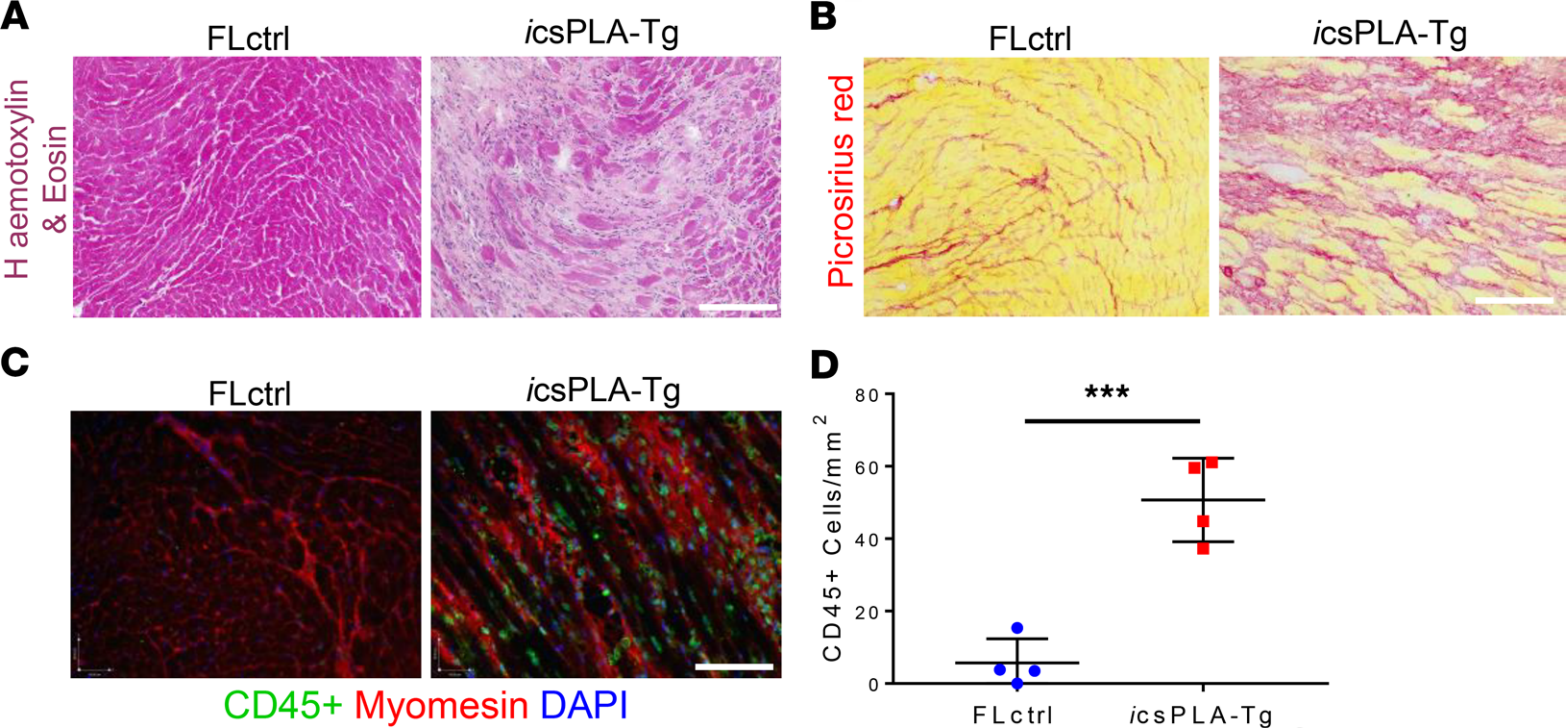
Induction of prelamin A in mouse hearts induces cardiac remodeling and inflammation. (**A**) H&E and (**B**) Picrosirius red staining showed myocardial disarray and fibrosis, respectively. (**C**) Immunoflourescence micrographs showing CD45^+^ cells were evident in *i*csPLA-Tg myocardium. Scale bars: 30 μm. (**D**) Quantification of CD45^+^ cells showing an increase in leukocyte population in *i*csPLA-Tg myocardium. Values are mean ± SD. *n* = 4 males/group. Unpaired student’s *t* test was performed. ***P < 0.001.

To this point, our model has tested constitutive accumulation of prelamin A, mostly relevant to inherited/genetic cardiomyopathies. Because HIV and associated cardiovascular diseases are predominantly acquired later in life, we wanted to mimic this by testing our model using an inducible MerCreMer system in adult mice and tracking disease onset by monitoring mice and performing serial echocardiography (Figure 10A). We induced mice at 34 ± 3 weeks of age with tamoxifen, which led to NE accumulation of prelamin A (Figure 10B). Median survival for csPLA-Tg mice was 65 days after injection (Figure 10C). While body weight remained consistent (Figure 10D), ejection fraction was significantly attenuated from 6 weeks onward, and left ventricular end-systolic volume was significantly increased at 8 weeks (Figure 10E). Moreover, histological examination revealed that, similar to constitutive expression of prelamin A, hearts were fibrotic and subjected to myocardial disarray (Figure 11, A and B). Importantly, CD45^+^ leukocyte infiltration was observed, indicating that inflammation was also evident, consistent with the phenotype observed in patients with acquired HIV cardiomyopathy (Figure 11, C and D).

## Discussion

### Accumulation of prelamin A occurs in genetic and HIV-associated cardiomyopathy.

To date, the role of prelamin A in the setting of cardiomyopathy has been insufficiently studied. Here, we investigated a cohort of patient samples, for which the primary diagnosis was idiopathic DCM. We showed consistent prelamin A accumulation in 1 DCM sample and sporadic accumulation in all other samples, including NF controls. Although sequencing of this 1 sample could not be performed, the consistent detection of prelamin A suggested that there may be a genetic basis for the disease in this patient. Additionally, this accumulation is likely to cause and/or exacerbate cardiomyopathy, as evidenced by our study of a potentially novel csPLA-Tg mouse line in which severe cardiac dysfunction was observed. We also present compelling evidence showing prelamin A accumulation in HIV-associated cardiomyopathy, since all samples tested showed elevated prelamin A abundance and nuclear morphology defects. A feature of HIV cardiomyopathy is inflammation and, consistent with a role for prelamin A in this, both constitutive and inducible mouse models of prelamin A accumulation developed extensive myocardial inflammation. In the constitutive model, we also observed myocardial senescence, and mechanistically, we linked the inflammatory response to modulation of NF-κB signaling, via activation of ATM. However, epigenetic changes may also be more important when considering early initiating mechanisms.

### Prelamin A accumulation causes inflammageing of the myocardium in mice.

Though inflammation is known to occur during and after ischemic events and later in heart failure (34), it is less commonly described in the progression to DCM. However, HIV-associated cardiomyopathy is strongly associated with an inflammatory response in the myocardium (35, 36). Recent reports show that highly active antiretroviral therapy (HAART) exposure in perinatal cases of HIV ultimately benefits heart function compared with patients from the pre-HAART era (37), for whom opportunistic infections (38) and potential incorporation and replication of HIV into CMs were problematic (39). Nevertheless, a decline in cardiac function occurs when compared with the normal population, and HAART therapies may contribute to this. NRTIs, which prevent replication of HIV, also inhibit transcription of mitochondrial DNA and have been shown to cause cardiac dysfunction in mice (25, 26). Our study presents evidence that inflammation is the key outcome of prelamin A accumulation in CMs, and this is also a relatively unique phenotype of HIV-associated cardiomyopathy. Though not subjected to rigorous interrogation of inflammatory pathways, global *Zmpste24^–/–^* mouse myocardium also showed leukocyte infiltration, further supporting a role for prelamin A toxicity in driving inflammation (40). In contrast, inflammation has not been reported in other *Lmna* mouse models of cardiomyopathy. Moreover, CM-specific overexpression of WT lamin A in mice showed no phenotypic effect or impact on survival, implying that processing mechanisms are able to cope with an increase in prelamin A concentration (41). In this context, it appears that accumulation of prelamin A, which cannot be processed, is pathogenic.

We provide evidence that the trigger for inflammation is likely linked to myocardial senescence. While expression of γ-H2AX, the most commonly used marker for DNA damage, was inconsistent in csPLA-Tg myocardium, ATM — which signals downstream of γ-H2AX — was persistently phosphorylated at 4 weeks. We observed that p16 and p21 mRNA was increased, along with mRNA of proinflammatory cytokines TNF-α, Icam1, Cxcl1, and Ccl2, suggesting that myocardium in these mice exhibit the SASP. We were able to further substantiate this by the detection of SA–β-Gal in csPLA-Tg myocardium. Furthermore, these data concur with previous work showing that, when prelamin A accumulates in vascular smooth muscle cells, activation of the SASP occurs (42).

### Early loss of repressive histone marks indicates that gene expression pathways are initiators of pathogenesis.

Global *Zmpste24^–/–^* mice suffer from systemic inflammation arising from noncanonical ATM-dependent NF-κB signaling (32), and we showed that this pathway was activated locally in csPLA-Tg hearts at 4 weeks, making it likely that increases in SASP factors are caused by activation of NF-κB. However, the absence of NF-κB signaling at 2 weeks suggests that this inflammatory pathway propagates rather than initiates disease mechanisms. Therefore, we speculated to other mechanisms for disease genesis. One of these was based on the mechanical hypothesis of lamin dysfunction. We reasoned that susceptibility to mechanical stress, which is continual and repetitive in the heart, might lead to structural defects in the NE and cytoskeleton. However, in the main these defects were not observed until 4 weeks, meaning this was unlikely. *Myh6* and *Myh7* mRNA encoding α- and β-MHC, respectively, did change at 2 weeks and may indicate early changes to myofilament structure; they may also reflect global changes in transcriptional activity as preceding structural defects. Therefore, we speculate that epigenetic changes may prime the tissue for disease, since chromatin displacement and loss of H3K9me3 were observed in 2-week-old mouse hearts. The mechanisms downstream of this remain to be determined but may involve misregulation of the polycomb repressive complexes (43), known to control senescent genes, such as those encoding p16 and p21, and potentially induce SASP independently of DNA damage pathways (44).

### Study limitations.

Due to difficulties in obtaining human tissue, we do not have the relevant control groups (e.g., HIV PI–naive) and/or alternate therapies (e.g., NNRTIs) to fully conclude that HIV PIs are the cause of prelamin A accumulation, though based on current literature, it is unlikely that these groups of patients would experience prelamin A accumulation.

Our constitutive mouse model was highly malignant and, as such, does not perhaps faithfully model the situation in cardiomyopathy, which tends to be more progressive in nature, though the *LMNA* cardiomyopathies are among the most severe. However, our inducible model was far less severe and represented a more progressive disease; it is also relevant to HIV associated cardiomyopathy, which are likely to be propagated by the accumulation of prelamin A, where HIV PIs with high binding affinity to ZMPSTE24 are still being used for treatment. However, one key difference is the model we used relied on mutating the site of ZMPSTE24 cleavage and, therefore, does not fully model the problem in HIV-associated cardiomyopathy, where a *Zmpste24*-KO or HIV PI pharmacological intervention would be interesting for further study. Despite this, our study shows in a reductionist manner that prelamin A is a toxic mediator of disease in cardiac tissue, indicating that disease is likely to be mediated by the accumulation of prelamin A specifically rather than any divergent targets or pathways, such as the declogging of translocons — the only other known and recently discovered function of ZMPSTE24 (45–47).

### Conclusions.

In summary, we have identified a potentially novel role for prelamin A in HIV-associated and dilated cardiomyopathies. Accumulation of prelamin A has catastrophic consequences for the integrity of the myocardium, resulting in an inflammageing phenotype and subsequent loss of contractility. While targeting inflammation potentially via ATM activity may prove useful for patients with established DCM owing to prelamin A accumulation, the immediate translational aspect of this study lies in the implications for the treatment of HIV-associated cardiomyopathy patients, for whom a change of therapy may have a beneficial outcome in the clinic. Elegant biochemistry performed by Robinson and colleagues provided compelling evidence that HIV PIs bind and block activity of ZMPSTE24 (48). Moreover, they were also able to show a rank order of affinity to ZMPSTE24 of HIV PIs currently available for use: lopinavir > ritonavir > amprenavir > darunavir. Of these, darunavir was shown not to bind *ZMPSTE24* at all, which confirms earlier work (49). Many HIV^+^ patients suffering from cardiac symptoms are not currently using darunavir in their HAART regimes (Table 1). Adjusting HAART regimes to incorporate darunavir and other HIV PIs with low affinity to ZPMSTE24 may reduce prelamin A accumulation and provide therapeutic benefit for patients suffering from symptoms of HIV-associated cardiomyopathy. Moreover, many therapeutic regimes are now beginning to move away from a HIV PI backbone, in favor of non-NRTIs (NNRTIs) (e.g., rilpivirine), and such an approach should be considered in patients with cardiac side-effects who are still receiving high doses of HIV PIs.

## Methods

Supplemental methods can be found online with this article.

### Generation of csPLA-Tg mice.

This mouse model was commissioned from Taconic-Artemis with the aim to devise a transgenic system to assess the in vivo effects of uncleavable prelamin A overexpression. We performed site-directed mutagenesis of the human *LMNA* gene at leucine 647 and replaced it with arginine (*LMNA*-L647R). This corresponds to the cleavage site for *ZMPSTE24* and blocks cleavage. The system involved recombinase-mediated cassette exchange (RMCE) of the Rosa26 gene whereby *LMNA-*L647R cDNA was inserted into an exchange vector containing a neomycin resistance gene, a strong CAGGS promoter sequence, and a STOP cassette flanked by loxP sites. Electroporation into the embryonic stem (ES) cells of C57BL/6 mice led to site-specific recombination by the recombinases F3 and FRT. Neomycin-resistant clones that had undergone RMCE were selected. After administration of hormones, superovulated BALB/c females were mated with BALB/c males. Blastocysts were isolated from the uterus at 3.5 days after coitum (dpc) for microinjection. Blastocysts were placed in a drop of DMEM with 15% FCS under mineral oil. A flat tip, piezo actuated microinjection-pipette with an internal diameter of 12–15 μm was used to inject 10–15 targeted C57BL/6NTac ES cells into each blastocyst. After recovery, 8 injected blastocysts were transferred to each uterine horn of 2.5 dpc, pseudopregnant NMRI females. Chimerism was measured in chimeras (G0) by coat color contribution of ES cells to the BALB/c host (black/white). Highly chimeric mice were bred to strain C57BL/6 females. Recombination by mating with mice carrying cre-recombinase under the control of the myosin light chain 2 ventricular (MLC2v) promoter led to removal of the STOP cassette, allowing expression of uncleavable prelamin A in nuclei of CMs of affected offspring. These mice were called csPLA-Tg mice. They were compared with mice expressing the transgene but retaining the lox P sites, termed FLctrl. For investigation of inducible prelamin A expression, breeding was performed with mice expressing cre-recombinase flanked by fragments of the estrogen receptor (MerCreMer), which allowed tamoxifen inducible expression also under the control of the MLC2v promoter and were termed inducible csPLA-Tg (*i*csPLA-Tg). Induction was performed by 3 once-per-day i.p. injections of 20 mg/kg of tamoxifen dissolved in peanut oil. Genotyping was performed by PCR on ear tissue DNA using primers designed to detect both the WT Rosa26 (377 bp) and modified Rosa26 (200 bp) loci (forward, 5′ - GTGGATGCTGAGAACAGGC - 3′; reverse, 5′ - TCCACCTGGTCCTCATGC - 3′) and also for the expression of cre-recombinase (forward, 5′ - TGCCAGGATCAGGGTTAA- 3′; reverse, 5′ - CCCGGCAAAACAGGTAGTTA - 3′). Cycling parameters were the following: initial denaturation 95°C, 1 minute; denaturation 95°C ,30 seconds (repeated for 35 cycles); annealing 60°C, 30 seconds (repeated for 35 cycles); extension 72°, 1 minute (repeated for 35 cycles); final extension 72°C, 10 minutes; cooled to 4°C indefinitely. All csPLA-Tg mice used in the study were heterozygous for the transgene and generated on a C57BL/6 background. Mice were kept in individually ventilated cages (IVC) at the biological services unit (BSU) at the Maurice Wohl Clinical Neuroscience Institute, King’s College London, fed a standard chow diet, and kept on a 12-hour light/dark cycle. Male and female mice were used in this study.

### Lmna^–/–^ mice.

A heterozygous breeding colony of mice with a null allele of *Lmna* (50) was established to obtain *Lmna*^−/−^, *Lmna*^−/+^, and *Lmna*^+/+^ (WT). Mice were genotyped by PCR on genomic tail DNA using the Manual ArchivePure DNA Purification Kit (5 Prime) using the following primers: forward, 5′ - CGATGAAGAGGGAAAGTTCG - 3′; mutant-specific reverse, 5′ - GCCGAATATCATGGTGGAAA - 3′; WT-specific reverse, 5′ - CCATGGACTGGTCCTGAAGT - 3′. Cycling parameters were the same as for csPLA-Tg genotyping. PCR produced a 750-bp amplicon from the mutated allele and a 520-bp amplicon from WT. Mice were housed at the BSU at New Hunts House, King’s College London, fed a standard chow diet and kept on a 12-hour light/dark cycle.

### Murine echocardiography.

Echocardiography was performed using a Vevo 2100 imaging system with a 30 MHz linear transducer especially designed for small animal studies (VisualSonics). Echocardiography was performed with 5% isoflurane fast induction of anesthesia followed by maintenance of 1%–1.5% isoflurane anesthesia for 4-week-old mice and 2.5% isofluorane for 2-week-old mice, which was vaporized in 100% oxygen delivered at 1.5–2 L/min. Heart rate was kept at about 400–450 beats per minute while respiratory rate was ~100 breaths per minute. Body temperature was ~36.5°C ± 1°C.

### Murine cardiac MRI.

Cardiac MRI was performed at the James Black Centre, King’s College London on a 7 Tesla (T) horizontal MR scanner (Varian Inc.) with mice in the prone position. The gradient coil had an inner diameter of 12 cm; the gradient strength was 1,000 mT/m (100 G/cm), and rise time was 120 ms. A quadrature transmit/receive coil (RAPID Biomedical GmbH) with an internal diameter of 39 mm was used. Anesthesia was maintained with 1.5% isoflurane/98.5% oxygen, and body temperature was maintained at 37°C using a warm air fan (SA Instruments). The ECG was monitored by means of 2 metallic needles placed s.c. in the front paws. A pressure transducer for respiratory gating was placed on the animal abdomen. To synchronize data acquisition with the ECG and to compensate for respiratory motion, simultaneous ECG triggering and respiration gating (SA Instruments) were applied. Functional and volumetric parameters were achieved following a multislice Cine-FLASH acquisition (51). These parameters were used: FOV = 25 × 25 mm^2^, slide thickness = 1 mm, matrix size = 128 × 128, 9–10 frames/cycle, 9 slices, flip angle =40°, cardiac cycle = 120 ± 30 ms, number of averages (number of signal averages or number of excitations employed to reduce signal to noise ratio) = 3, acquisition time = approximately 8 minutes. Functional and volumetric parameters were calculated from CineFLASH images, and areas of contrast enhancement were calculated using a semiautomated in-house–developed computer software program (King’s College London, ClinicalVolumes).

For detection of myocardial scarring by T1 mapping (52), anesthetized mice were subject to MRI before and 25 minutes after i.p. administration of 0.75 mmol/kg of gadofosveset trisodium (Ablavar, Lantheus Medical Imaging), a gadolinium-based contrast agent. An ECG-triggered, single slice, Look-Locker acquisition was used for T1 mapping and to measure R1 values of the myocardium. The slice was selected in the middle of the heart. Imaging parameters included FOV = 25 × 25 mm^2^; slice thickness = 1 mm; matrix size = 128 × 128, 3 phases/cycle, total of 30 phases, 1 slice, flip angle = 10°, repetition time (TR) = 2700 ms, effective TR (TReff) = approximately 40 ms ([cardiac cycle]/[3 phase/cycle]); echo time (TE) = 2 ms; cardiac cycle = 120 ± 20 ms; number of averages = 1; acquisition time = approximately 13 minutes. T1-weighted sequences were analyzed to assess the R1 values of the myocardium.

Look-Locker T1 mapping resulted in 30 images (3 per cardiac cycle) from which R1 values of myocardium were calculated using an exponential 3 parameter fit (A-B*exp [–TI/T1*]) with subsequent T1 correction (OriginLab Corp.). Where A and B are fitting parameters relating to the equilibrium magnetization and type of preparation, TI represents inversion delays and the asterisks denote ‘effective’ or ‘observed’ values.

### TEM.

Mice were injected i.p. with heparin (5000 units/kg body weight). This was followed by i.p. injection of 50mg/kg body weight of sodium pentobarbital to induce terminal anesthesia. The chest cavity was opened and secured with a hemostat. The LV was injected with a needle connected via a pump to a reservoir of prewash buffer. Flow rate of the pump was adjusted as to perfuse mouse heart at a pressure between 90 and 100 mmHg with prewash buffer. Prewash buffer was a standard physiological tyrode solution containing 10 mM BDM (2, 3 butanedione monoxime [Sigma]) to arrest the heart in diastole and 2.5% PVP (polyvinylpyrrolidone [Sigma]) to replace the protein content of blood, thereby maintaining colloidal pressure and preventing hemorrhage at vascular sites in the heart. Following prewash, the hearts were perfused with fixative solution containing 2% glutaraldehyde (Agar Scientific) and 2% paraformaldehyde (Electron Microscopy Services) until 20 ml of fixative had been perfused. Hearts were dissected, and the mid-LV was isolated and cut for further processing. Samples were dehydrated through a graded series of ethanol washes and embedded in epoxy resin. Semithin sections (0.2 μm) were stained with Toluidine blue for light microscopy examinations and were used to guide sampling for TEM studies. Thin sections (0.09 μm) were collected on 150 mesh copper grids and double-stained with uranyl acetate and lead citrate for examination under TEM (H7650, Hitachi).

Human HIV^+^ samples were fixed in 2% glutaraldehyde in a 0.1 M phosphate buffer, at pH 7.3, postfixed in osmium tetroxide, and processed following a standard schedule for embedding in Epon Resin (Hexion). Semithin sections (0.2 μm) were stained either with Azur II (Sigma) or basic fuchsin solutions and mounted with permount medium. Ultrathin sections (70–80 nm) were stained with uranyl acetate and lead hydroxide. A Jeol 1400 plus TEM was used for observation and photographic analysis.

### Human echocardiography.

Single-center echocardiography was performed using a Philips epic ultrasound machine. Echocardiographic parameters were determined according to established criteria (53). In particular, ejection fraction was calculated in the apical 4- and 2-chamber views from 3 separate cardiac cycles using the modified Simpson’s method, and left ventricular end-diastolic diameter was measured in long-axis and short-axis view.

### Statistics.

All in vivo and ex vivo data of csPLA-Tg vs. FLctrl mice were analyzed using the a 2-way ANOVA without repeated measures with Sidak’s post hoc test for direct comparison of age-matched groups. Student’s unpaired 2-tailed *t* test was applied when only 4-week-old mice were compared. Where unequal variances were observed, Welch’s correction was applied. For the analyses of body weights over time and serial echocardiography, 2-way ANOVA with repeated measures was selected with Sidak’s post hoc test for comparison of groups at specific time points of the series. Kaplan-Meier survival curves were assessed by the log-rank Cox-Mantel test. Values were expressed as means ± SD. *P* < 0.05 was considered significant. Tests were performed in Excel (Microsoft) or Prism (GraphPad).

### Study approval.

This study complies with the declaration of Helsinki. Human DCM specimens were obtained from the Sydney Heart Bank (Hospital Research Ethical Committee approval H03/118; University of Sydney ethical approval 12146) and from Papworth tissue bank in Cambridge, United Kingdom, and used in accordance with ethical guidelines of King’s College London (REC reference 13/LO/1950) and the current United Kingdom law. Studies involving HIV-associated cardiomyopathy patient endomyocardial biopsies were approved by the Ethics Committee of La Sapienza University (Rome, Italy) and performed because, despite good control of HIV infection, patients were experiencing progressive cardiomyopathy. All patients gave informed consent. All animal procedures were performed in accordance with the Guidance on the Operation of the Animals (Scientific Procedures) Act, 1986 (UK Home Office).

## Author contributions

DB and CMS conceived and designed the study, cowrote the manuscript, and supervised the work. DB also conducted experiments and analyzed data. AF, RV, CC, and MAR performed all studies concerning HIV patients and corresponding samples. RH performed animal husbandry, morphometry, and tissue collection. SMA performed IHC and data analysis. GVB prepared the samples for electron microscopy. AP performed and analyzed the murine cardiac MRI. PSZ supplied the *Lmna^–/–^* mice. EE and CGDR sourced and supplied the DCM explants and nonfailing donor heart tissue. AMS cosupervised the study and contributed to manuscript preparation.

## Supplementary Material

Supplemental data

## Figures and Tables

**Table 1:**
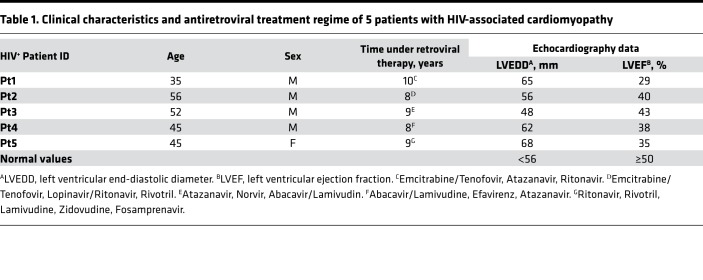
Clinical characteristics and antiretroviral treatment regime of 5 patients with HIV-associated cardiomyopathy

## Version 1 10/17/2019

In-Press Preview

## Version 2 11/14/2019

Electronic publication

## Version 3 11/14/2019

Sadia Ahmad's name was updated.
