# Supplementary material for: Prelamin A mediates myocardial inflammation in dilated and HIV-associated cardiomyopathies
Source: JCI Insight. 2019 Nov 14;4(22):e126315. doi: 10.1172/jci.insight.126315 (PMC6948859; doi:10.1172/jci.insight.126315)

Supplemental Materials for

'Prelamin A mediates myocardial inflammation in dilated and HIV associated cardiomyopathies'

Authors: Daniel Brayson, PhD[1]; Andrea Frustaci, MD[2,3]; Romina Verardo, PhD[3]; Cristina Chimenti, MD PhD[2,3]; Matteo Antonio Russo, MD[4]; Robert Hayward, BSc[1]; Sadia Munir Ahmad, MSc[1]; Gema Vizcay-Barrena, PhD[5], Andrea Protti, PhD[6]; Peter S. Zammit, PhD[7], Cristobal G. dos Remedios, PhD[8]; Elisabeth Ehler, PhD[1,7]; Ajay M. Shah, MD[1]; Catherine M. Shanahan, PhD[1]

Addresses:

[1]School of Cardiovascular Medicine and Sciences, King's College London BHF Centre for Research Excellence, London, UK

[2]Department of Cardiovascular, Nefrologic, Anestesiologic and Geriatric Sciences, La Sapienza University of Rome, Italy.

[3]National Institute for Infectious Diseases IRCCS 'L. Spallanzani', Rome, Italy

[4]MEBIC Open University San Raffaele and IRCCS San Raffaele Pisana, Laboratory of Molecular and Cellular Pathology, Milan, Italy

[5]Centre for Ultrastructural Imaging, Guy's Campus, King's College London, London, UK

[6] Department of Imaging, Lurie Family Imaging Center, Center for Biomedical Imaging in Oncology, Dana-Farber Cancer Institute, Harvard Medical School, Boston, Massachusetts, USA,

[7]Randall Centre for Cell and Molecular Biophysics, King's College London, UK

[8]Department of Anatomy, Bosch Institute, University of Sydney, Sydney, NSW, Australia

Working Title: Brayson et al., Accumulation of prelamin A in cardiomyopathy

Address for correspondence: 3rd Floor James Black Centre
125 Coldharbour Lane
King's College London
London SE5 9NU

Telephone: +442078485223

Email: daniel.brayson @kcl.ac.uk

Supplemental Methods

Morphometry

Mice were subjected to observation and weighed regularly. When it became apparent that the phenotype of the csPLA-Tg mice was failure to thrive and premature death, it was decided that when the weight of a mouse fell below 85% of its FLctrl littermates, it was deemed to be suffering the maximum threshold of pain permitted on the project license and was culled. Post-mortem morphometry analysis was performed using heart weight and tibia length.

Indirect immunofluorescence of cryopreserved tissue

Tissue was removed from storage at -80°C and warmed to -20°C for approximately 1 hour. Tissue was then mounted onto the stage of a cryostat with OCT compound and allowed to equilibrate to the ambient temperature of the cryostat for 5 min; for cutting heart tissue, the stage temperature was set to -22°C and the knife was set to -20°C. The sections were then cut to 10 μm thick and mounted onto high quality polylysine or superfrost slides. The sections were allowed to air dry for approximately 1 hour and then stored at -80°C. For staining, sections were allowed to dry completely and fixed by immersion, in a Coplin jar, in pre-chilled 100% methanol at -20°C for 5 min or 4% formalin for 10 min RT. Sections were washed 2x5 min in PBS. Sections were permeabilised in 0.5% NP-40 for 3 min RT and washed 3x5 min in PBS. Sections were blocked in 3% serum originating from a species to which the antibody was not raised against for 1 hour RT. Primary antibody was diluted appropriately in blocking solution applied to the sections and incubated in a humidified chamber overnight at 4°C. Sections were washed 3x5 min in PBS and secondary antibody conjugated to a fluorophore was diluted as appropriate, applied to the sections and incubated in the dark in a humidified chamber for 1 hour RT. DAPI was added 1:10,000 for 5 min at the end of the incubation for visualisation of nucleic structures. Sections were washed 3x5 min in PBS in the dark and then mounted in mowiol mounting media and allowed to dry in the dark overnight. Source of antibodies dilutions can be found in Supplemental Table 3.

Microscopy

Immunohistochemical and general tissue staining was analysed by bright-field light microscopy using an Axioskop 2 microscope (Carl Zeiss MicroImaging, NY). Immunofluorescence was analysed using wide-field fluorescence microscopy (IX81 microscope, Olympus). Where images captured were subjected to quantitative analysis, this was performed by counting by eye, or semi automated with the use of selection and measuring tools made freely available by ImageJ software. All massays were performed at the James Black Centre, King’s College London and in each case the investigator performing the analysis was blinded to group assignment. Images for publication were captured using a Leica SP5 confocal microscope (Leica Microsystems, UK). Confocal images were processed in Photoshop CS3 (Adobe) and minimal adjustments to brightness and contrast were made where necessary, e.g. when images were deemed too dark for comfortable viewing.

Immunoblotting

Cryopreserved heart tissue was homogenised by placement into a pre-liquid nitrogen cooled stainless steel chamber followed by crushing administered by a pre-liquid nitrogen cooled stainless steel rod and rubber mallet. 200 µl lysis/sample buffer (3.7 M urea, 134.6 mM Tris pH 6.8; 5.4% SDS; 2.3% NP-40; 4.45% beta-mercaptoethanol; 4% glycerol and 6 mg/100 ml bromophenolblue) was added. And frozen pellets placed into Eppendorf tubes. The tubes were heated to 85°C for 5 minutes. A chamber was prepared with an SDS gel of 10% acrylamide concentration. 20µl was then loaded into each well of the gel. The proteins were separated at 150 mV. Transfer was achieved in wet conditions. The proteins were subject to electrophoretic transfer onto PVDF membranes that had been incubated in methanol for 20 seconds and then washed in distilled water. The transfer conditions were 65 Amps at 4°C ON. The membrane was blocked for 1 hour with 5% Milk in TBS-T. Primary antibodies were added (Table 2.2) with TBS-T/5% milk or TBS-T 5% bovine serum albumin and incubated overnight at 4°C. The membrane was washed 3x5 min with TBS-T. The blots were then incubated in secondary antibody coupled to horseradish peroxidise for 1 hour at room temperature or secondary antibody conjugated to a fluorophore or horse radish peroxidase for 1 hour. The blots were again washed with

TBS-T and bound antibody signal was detected by an Odyssey® scanning detection system (LI-COR Biosciences, U.S.A), or by enhanced chemilumiescence substrate (ECL plus, GE Healthcare, USA) and exposure to film. Western blots were quantified using ImageStudio™ software (LI-COR Biosciences, U.S.A) and ImageJ. Immunoblotting of HIV associated cardiomyopathy tissue was performed using commercially available kit for whole-cell extraction from frozen tissue according to manufacturers instructions (Active Motif, USA, cat. no. 40010).

Quantitative (q)-PCR

2 µg RNA was diluted into 10 µl of double distilled (dd) H_2O. 0.3 µl Oligo dT primer, 0.3 µl random primer and 1µl dNTPs was added to this and heated to 65°C and then cooled to 4°C in a thermocycler. 4 µl 5x Buffer, 3.9 µl DEPC-treated H_2O and 0.5 µl reverse transcriptase (M-MLV) was added and samples were heated in a thermocycler to 25°C for 10 min, 37°C for 50 min and cooled to 4°C. After the reaction, the samples were diluted to 100 µl total volume for qPCR. qPCR was carried out using the ΔCt and standard curve methods. When the ΔCt method was used the primers had been pre-validated and were part of a standard protocol for the lab. 9 µl cDNA was added to 10 µl 2x Sybr green PCR master mix and 5 µmol of forward and reverse primers (Supp table. 4). Cycling parameters were 94°C for 15 seconds, followed by single-step annealing and extension at 60°C for 1 min (35 cycles). Reactions were carried out in a Corbett RotorGene-3000. The cycle threshold (Ct) was determined manually as an arbitrary point during the linear phase of amplification and mRNA expression was quantified as a ratio of GAPDH expression. mRNA expression of csPLA-Tg groups was expressed as fold change compared to FLctrl groups.

Haemotoxylin and eosin staining

Tissue samples were fixed in 4% PFA and dehydrated overnight. The samples were then embedded in wax blocks and cut to sections of 6 µM thickness and mounted on glass slides (superfrost plus, VWR International). Slides were baked at 60°C overnight. Tissues were then processed and rehydrated by sequential incubation in xylene and reducing concentrations of ethanol 100%, (2x3 min) 96%, 75%, 50%, 25% (1 min). After rehydration, slides were left under running tap water for 10 min. After

rehydration, sections were washed under running tap water for 5 min, and then placed in Harris' haematoxylin solution for 5 min. The sections were then washed under running tap water until the water ran clear followed by differentiation in acid alcohol. The sections were again placed under running tap water for 5 min and then stained with eosin solution for 3 min. The sections were dunked 5 times in tap water and then subjected to dehydration by sequential ethanol treatment: 25%, 50%, 75%, 96% (1 min each) 100%, (2x3 min) xylene, (2x5 min). Coverslips were mounted using DPX and allowed to dry overnight.

Picro-sirius red staining

Following rehydration, slides were left in ddH_2O for 5 min followed by a 30 second incubation in 0.2% phosphomolybdic acid. Slides were rinsed in ddH_2O and then left in 1% Sirius red solution for 90 min. Slides were washed 2x2 min in acidified ddH_2O (0.05% acetic acid) followed by a 15 min incubation in Picric acid. Slides were rinsed several times in ddH_2O followed by dehydration by sequential ethanol treatment: Glass coverslips were mounted with DPX and allowed to dry overnight.

Senescence associated β-Galactosidase assay

Hearts were resected from mice and immediately frozen and cryosectioned into 5mm thick slices and mounted onto superfrost slides. Sections were allowed to dry for 1-2 hrs. Samples were fixed in 1% formalin solution for 10 min RT. Senescence associated β-Galactosidase (SA-β-Gal) staining solution was freshly made and applied to each heart section so that they were generously submerged (approx. 50 μl per heart section). The slides were incubated in a humidified chamber overnight (approx. 16h) at 37°C. The slides were washed 2x5 min in PBS and once in 100% methanol for 2 min, the heart sections were then viewed and photographed under bright-field microscopy.

Cardiac troponin T (cTnT) Enzyme Linked Immuno-Sorbent Assay (ELISA)

Cardiac puncture under terminal anaesthesia was performed and blood was collected in an EDTA coated Eppendorf and spun at 13,000 g 4°C for 15 minutes to sediment red blood cells. Plasma was

collected and subject to ELISA for cTnT (Elabscience, USA) according to manufacturer's instructions.

TUNEL staining

Deparaffinised sections were digested in 20 µg/ml proteinase K solution at 37°C for 10 min and washed 3x5 min in PBS. Sections were then incubated in 3% H_2O_2 for 10 min RT to block endogenous peroxidase activity and washed 3x5 min in PBS. Sections were then pre-incubated in TdT reaction buffer for 10 min RT and then incubated in TdT reaction mixture for 1 hour at 37°C in a humidified chamber. Sections were then washed in stop wash buffer for 10 min RT and rinsed in PBS-T 3x5 min. Sections were incubated with streptavidin-HRP for 20 min RT. Sections were incubated in DAB chromagen solution for 1-10 min (until brown staining appeared) and left under running tap water for 5 min. Coverslips were mounted with DPX and allowed to dry for 2 hour or overnight (ON).

Supplemental Tables

Supplemental Table 1. Patient information for explanted DCM samples

Random ID	Assigned ID	Sex	Age	Clinical Diagnosis	Gene	Mutation	Ethnicity	Diabetes	Ejection fraction (%)	Biobank centre	Nature of source
2.059	DCM01	M	43	FDCM	NA	NA	NA	NA	NA	SHB	Explanted at transplantation
4.032	DCM02	M	31	DCM	NA	NA	NA	NA	NA	SHB	Explanted at transplantation
4.047	DCM03	F	63	FDCM	MYOM1	E247K	NA	NA	NA	SHB	Explanted at transplantation
4.066	DCM04	M	52	FDCM IHD	PKP2	I531S	NA	NA	NA	SHB	Explanted at transplantation
S99 1036	DCM05	NA	NA	DCM	NA	NA	NA	NA	NA	Papworth	Explanted at transplantation
TB11.1626	DCM06	M	45	DCM	NA	NA	White british	No	15	Papworth	Explanted at transplantation
TB11.1730	DCM07	M	41	DCM	NA	NA	White british	No	8	Papworth	Explanted at transplantation
TB12.0754	DCM08	M	43	FDCM	NA	NA	Asian british Indian	No	NA	Papworth	Explanted at transplantation
TB12.1094	DCM09	M	29	DCM	NA	NA	White british	No	NA	Papworth	Explanted at transplantation

TB13.0359	DCM10	M	24	DCM	NA	NA	White british	No	NA	Papworth	Explanted at transplantation
TB13.0992	DCM11	M	25	DCM	NA	NA	White British	No	NA	Papworth	Explanted at transplantation
TB13.1292	DCM12	M	50	DCM, NYHA calss III heart failure	NA	NA	Whit British	No	NA	Papworth	Explanted at transplantation
TB13.2060	DCM13	M	42	DCM	NA	NA	White British	No	NA	Papworth	Explanted at transplantation
TB13.2131	DCM14	M	64	DCM with biventricular dysfunction	NA	NA	NA	No	NA	Papworth	Explanted at transplantation
TB13.2163	DCM15	F	53	Restrictive Cardiomyop athy	NA	NA	White British	No	NA	Papworth	Explanted at transplantation
TB13.2277	DCM16	M	37	DCM	NA	NA	NA	NA	NA	Papworth	Explanted at transplantation
TB14.0606	DCM17	M	65	DCM with RV ischaemia	NA	NA	NA	No	NA	Papworth	Explanted at transplantation
TB14.1985	DCM18	M	34	DCM	NA	NA	White british	No	NA	Papworth	Explanted at transplantation
TB14.2015	DCM19	M	44	DCM	NA	NA	White british	Type II	NA	Papworth	Explanted at transplantation

TB15.0559	DCM20	F	31	DCM	NA	NA	Black british carribean	Type II	NA	Papworth	Explanted at transplantation
TB16.0638	DCM21	M	52	DCM	NA	NA	NA	No	NA	Papworth	Explanted at transplantation

1

Supplemental Table 2. Non-failing controls used in this investigation

Random ID	Assigned ID	Biobank centre	Nature of Source
S99 577	NF01	unused donor	unused donor
Ch1 5.089	NF02	SHB	unused donor
Ch1 5.138	NF03	SHB	unused donor
LV 7.004	NF04	SHB	unused donor
LV 7.012	NF05	SHB	unused donor
LV 7.060	NF06	SHB	unused donor
LV. 7.072	NF07	SHB	unused donor
LV 8.010	NF08	SHB	unused donor
LV NB 5.048	NF09	SHB	unused donor
S99 1309	NF10	unused donor	unused donor

SHB = Sydney Heart Bank

Supp Table 3. List of antibodies used in this investigation

Primary Antibodies	Source	Dilution (WB)	Dilution (IF)	Dilution (IHC)
CD3	Dako M7254	-	-	1:20
CD45	Abcam ab10558	1:1000	1:100	-
Cleaved Caspase3	Cell Signaling 9661	1:1000	-	-
Desmin	Dako M0760	1:100	-	-
Emerin	Leica Biosystems NCL-Emerin	1:500	-	-
GAPDH	Sigma-Aldrich G8795	1:1000	-	-
H3k9me3	abcam	-	-	1:1000
Lamin A/C (N-18)	Santa Cruz Biotechnology sc-6215	1:1000	-	-
Myomesin B4	Gift C/O EE (1)	-	1:100-200	-
Nesprin 2 N3	Homemade	1:1000	-	
γ-H2AX	Cell signalling 9718	1:1000	1:1000	-
p16	Cell signalling 4824		-	1:1000
p65	Santa cruz Biotechnology sc-372	1:500	1:200	-
Prelamin-A (C-20)	Santa Cruz Biotechnology sc-6214	1:200	1:1000	-
SUN 2	Abcam ab65447	1:100	-	-
**Secondary Antibodies**	**Source and cat number**	**Dilution (WB)**	**Dilution (IF)**	**Dilution (IHC)**
Anti-goat Alexa fluor 488.	Invitrogen A-11055	-	1:1000	-
Anti-goat Alexa fluor 568	Invitrogen A-11057	-	1:500	-
Anti-mouse Alexa 488	Invitrogen A11004	-	1:1000	-

Anti-mouse Cy3	Jackson Laboratories 715-150-150	-	1:1000	-
Anti-rabbit Alexa fluor 488	Invitrogen A-11011	-	1:1000	-
Biotinylated anti-goat	VECTASTAIN ABC kit (see reagents)	-	-	1:500
IRdye 800 Anti-goat	LICOR 926-32214	1:15000	-	-
IRdye 800 Anti-mouse	LICOR 926-32210	1:15000	-	-
IRdye 800 Anti-rabbit	LICOR 926-32211	1:15000	-	-
IRdye680 Anti-rabbit	LICOR 926-68073	1:15000	-	-

Supp Table 4. Primers used in this investigation for qPCR experiments

**Primers**	**Sequence (5’-3’)**
Nppa	F—CGTGCCCCGACCCACGCCAGCATGGGCTCC<br>R—GGCTCCGAGGGCCAGCGAGCAGAGCCCTCA
Nppb	F—AAGGGAGAATACGGCATCATT<br>R—ACAGCACCTTCAGGAGATCCA
Myh6	F—CAGAGATTTCTCCAACCCAGCTGCG<br>R—AGTCAGCCATCTGGGCGTCCG
Myh7	F—AGCAGCAGTTGGATGAGCGACT<br>R—CCAGCTCCTCGATGCGTGCC
GAPDH	F—CGTGCCGCCTGGAGAA<br>R—CCCTCAGATGCCTGCTTCAC
Ccl2	F—TTAAAAACCTGGATCGGAACCAA<br>R—GCATTAGCTTCAGATTTACGGGT
Icam1	F—GGCATTGTTCTCTAATGTCTCCG<br>R—TGTCGAGCTTTGGGATGGTAG
Tnf	F—CAGGCGGTGCCTATGTCTC<br>R—CGATCACCCCGAAGTTCAGTAG
Cxcl1	F—ACTGCACCCAAACCGAAGTC<br>R—TGGGGACACCTTTTAGCATCTT
Cdkn2a (p16)	Proprietary from Qiagen (Cat No. QT00252595)
Cdk1a (p21)	Proprietary from Qiagen (Cat No. QT00137053)

Reference

1. Grove BK, Kurer V, Lehner C, Doetschman TC, Perriard JC, and Eppenberger HM. A new 185,000-dalton skeletal muscle protein detected by monoclonal antibodies. *The Journal of cell biology.* 1984;98(2):518-24.

Full unedited gels for figure 1

Prelamin A

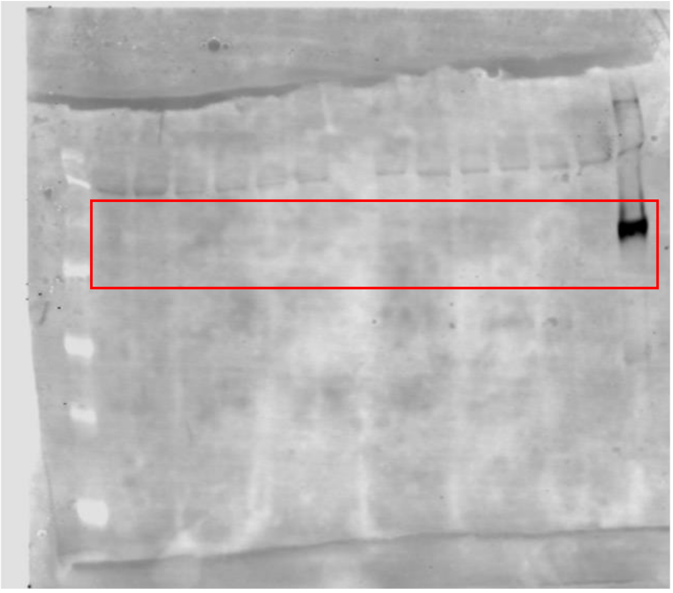

Ponceau S

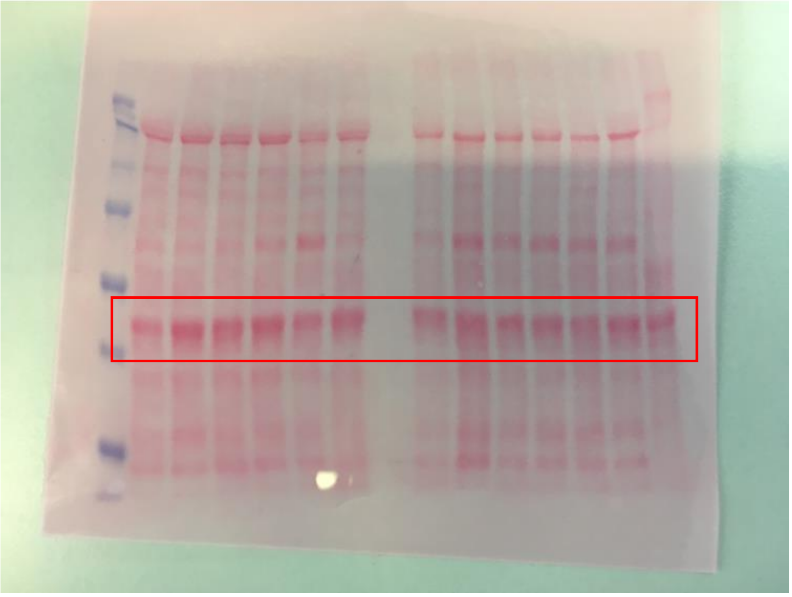

Full unedited gels for figure 2

Prelamin A

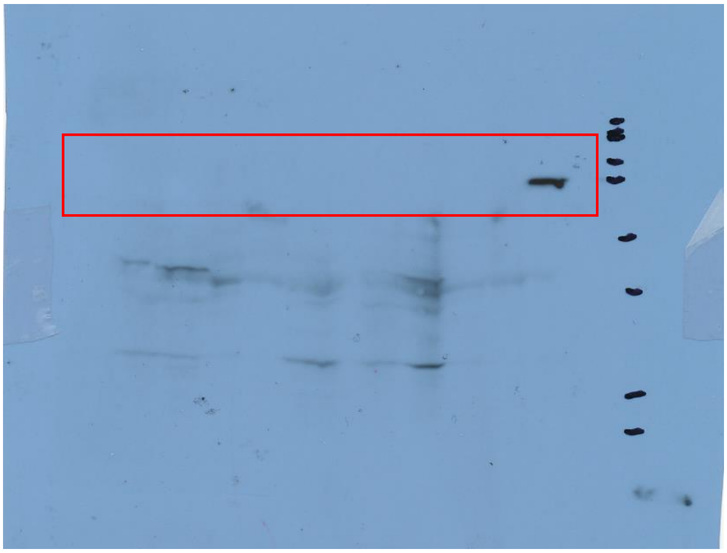

Beta actin

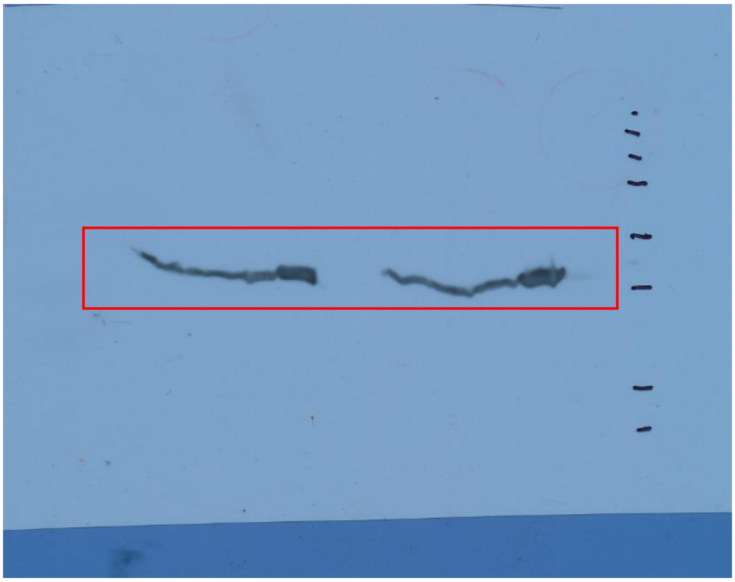

Full unedited gels for figure 3

Lamin A/C

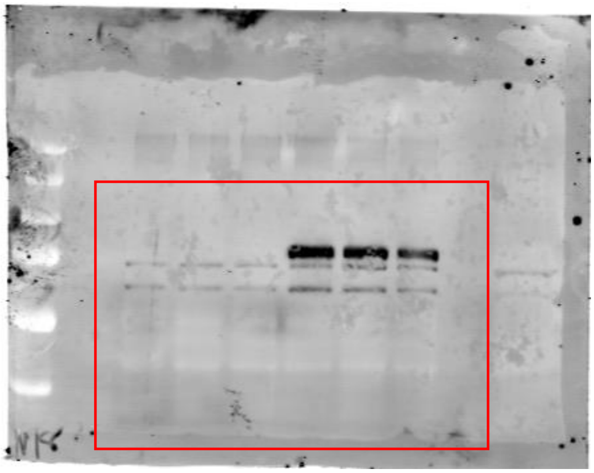

Caspase 3

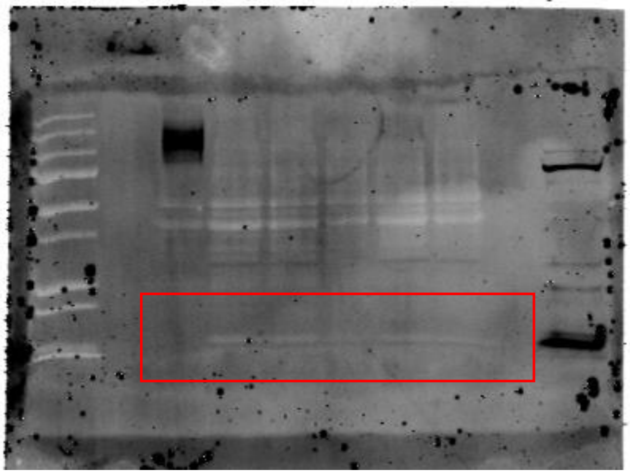

GAPDH

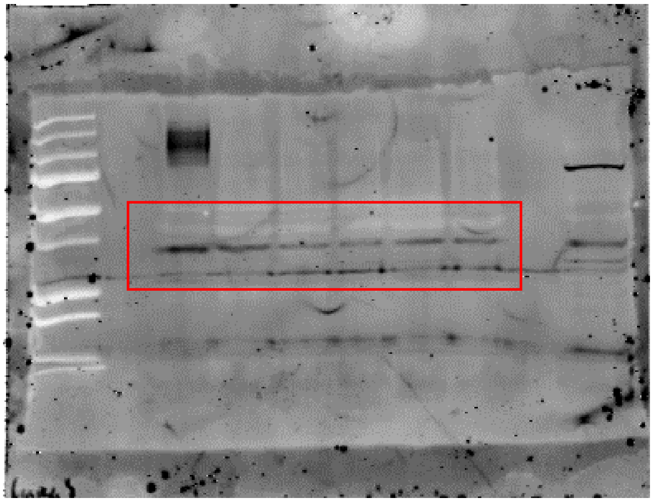

Full unedited gels for Figure 6

B.

p65

GAPDH

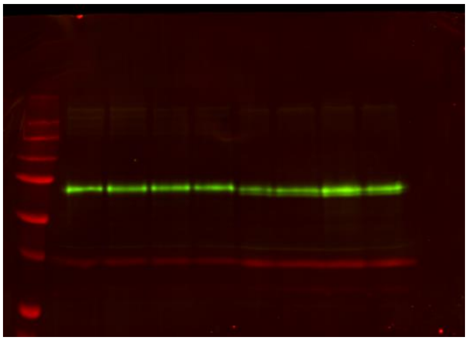

D cont…

Total ATM

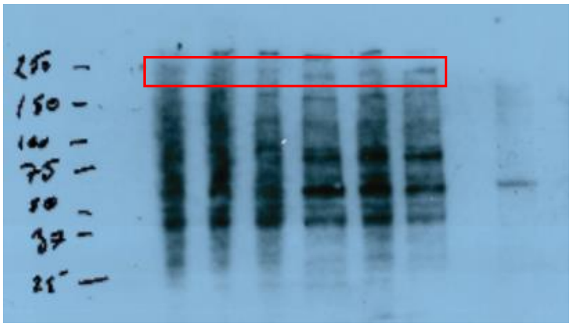

Phospho ATM

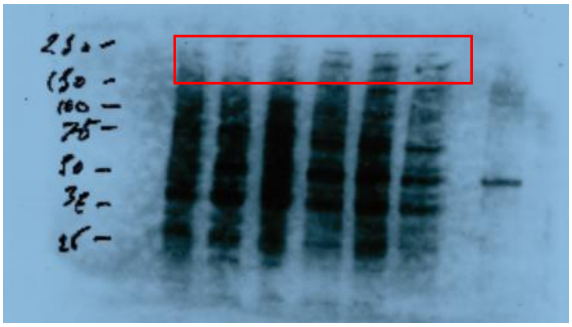

D.

p65

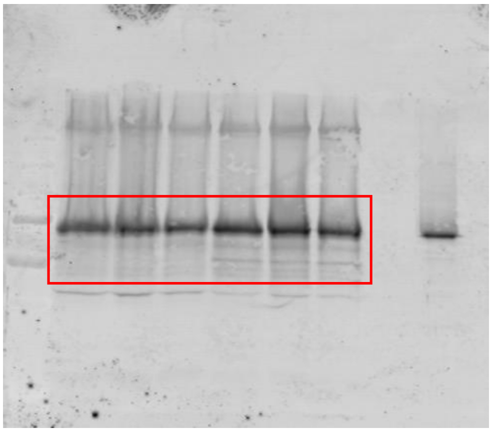

GAPDH

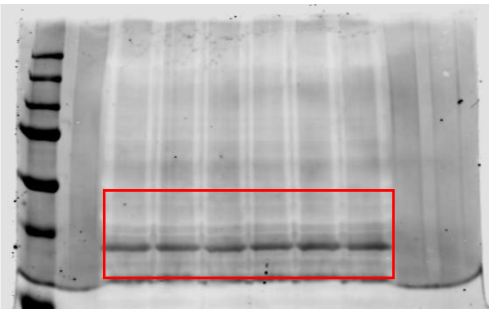

Total ikba

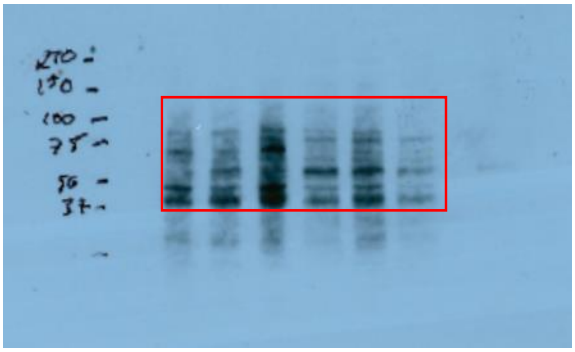

Phospho ikba

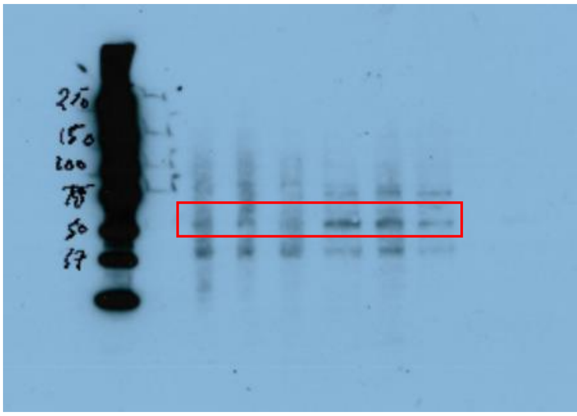

Full unedited gels for figure 7

Prelamin A

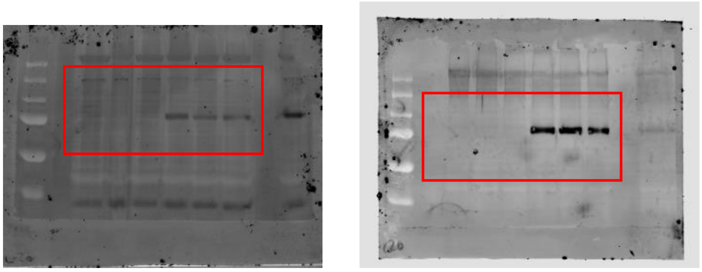

Lamin A/C

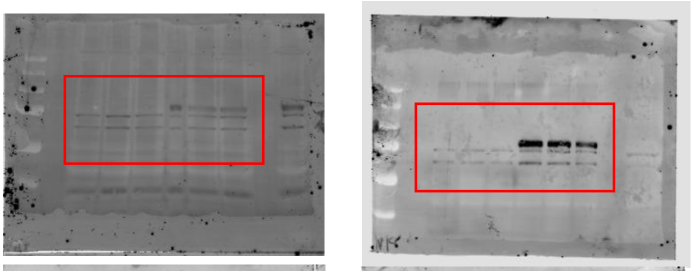

Emerin

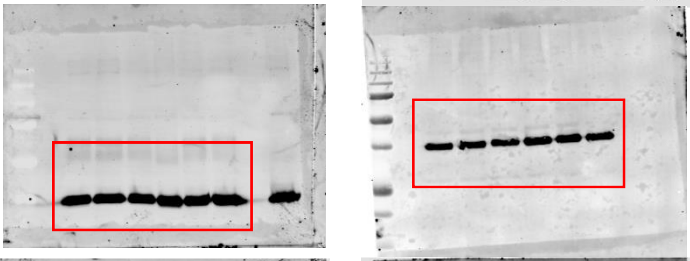

SUN2

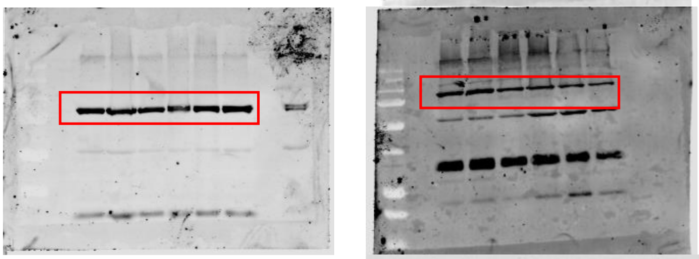

Nesprin 2

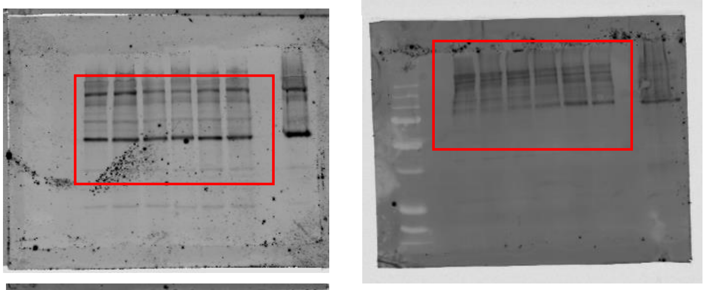

Desmin

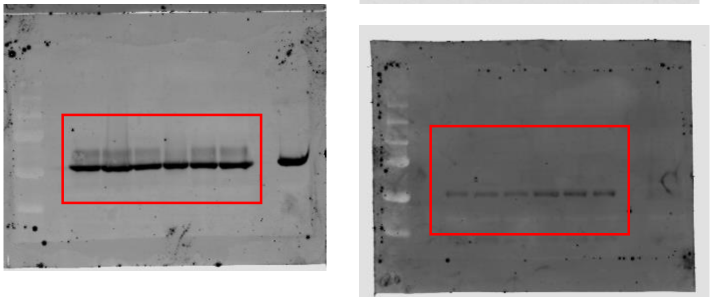

GAPDH

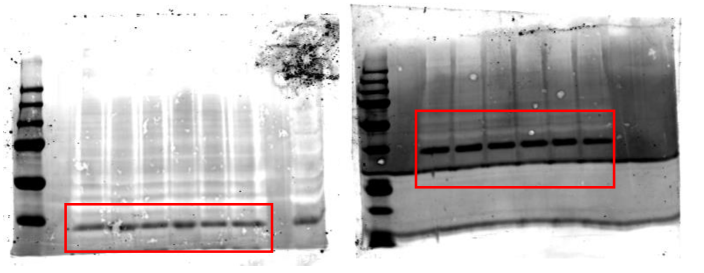

Full unedited gels for Figure 9

Prelamin A

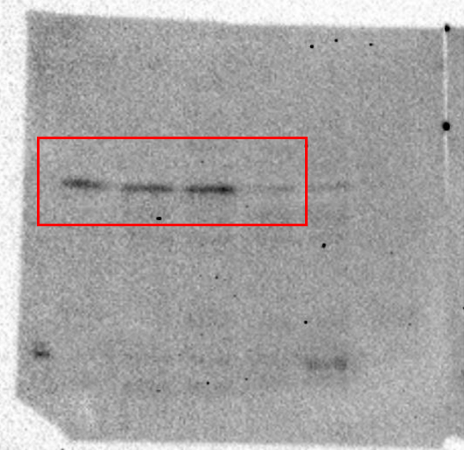

Ponceau S

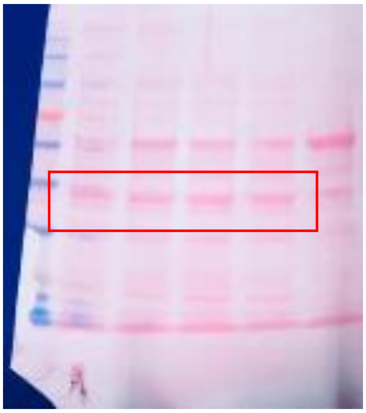

Supplement: Supplemental data [file jciinsight-4-126315-s230.pdf]
